# Chitosan and Chitin-Derived Biomaterials in Orthopedics: A Structured Narrative Review of Polymer Design, Quantitative Performance, and Clinical Translation

**DOI:** 10.3390/polym18131644

**Published:** 2026-07-01

**Authors:** Furkan Yapıcı

**Affiliations:** Department of Orthopedics and Traumatology, Erzincan Binali Yıldırım University, Erzincan 24100, Turkey; furkan.yapici@erzincan.edu.tr

**Keywords:** chitosan, orthopedic biomaterials, polymer platform, cartilage repair, bone regeneration, hydrogels, implant coatings, bone cement, intervertebral disk, clinical translation

## Abstract

Chitosan and chitin-derived biomaterials, including native chitosan and chemically modified derivatives, have been widely investigated across orthopedic tissue engineering, implant functionalization, infection control, local delivery, and interface repair, but the evidence is dispersed across heterogeneous formats and indications. This single-author structured narrative review synthesizes 258 unique publications and interprets chitosan through a polymer design, quantitative performance, and clinical translation framework. Literature was identified (January–May 2026) using PubMed/MEDLINE as the primary database, with targeted verification in Web of Science, Scopus, and Google Scholar; no formal risk-of-bias or certainty grading was performed. Chitosan was studied as scaffolds, hydrogels, coatings, nanoparticles, microspheres, fibers, bioadhesives, bone-cement additives, cartilage adjuncts, tendon-to-bone systems, and intervertebral disk biomaterials. The highest human clinical evidence supported BST-CarGel/chitosan–blood implant augmentation of knee marrow stimulation, where randomized, 5-year, and biopsy data favored structural repair over microfracture alone; most other applications—bone regeneration, coatings, osteomyelitis hydrogels, bone cements, tendon/rotator cuff systems, and disk biomaterials—remain preclinical or translational-preclinical. Chitosan should be interpreted as a tunable polymer platform, not a single material; translation requires chemistry-defined formulation, indication-specific mechanical qualification, clinically relevant comparators, and standardized reporting.

## 1. Introduction

### 1.1. Orthopedic Need for Multifunctional and Indication-Specific Biomaterials

Orthopedic tissue defects remain a major clinical and socioeconomic challenge because musculoskeletal tissues differ substantially in vascularity, cellularity, mechanical loading, intrinsic healing capacity, and susceptibility to infection. Bone can regenerate under favorable conditions, but segmental defects, fracture nonunions, revision arthroplasty defects, spinal fusion defects, tumor-related bone loss, osteomyelitis-associated cavities, and critical-size defects frequently exceed the biological repair capacity of native tissue [1]. Cartilage and osteochondral defects represent a different problem: articular cartilage is avascular, aneural, sparsely cellular, and mechanically demanding, which limits intrinsic repair and often leads to fibrocartilaginous rather than hyaline-like repair after marrow-stimulation procedures [2]. Tendon-to-bone, ligament, rotator cuff, and enthesis injuries are similarly difficult because the native graded fibrocartilaginous interface is rarely restored after surgical repair [3]. Intervertebral disk degeneration adds another challenge because the disk is an avascular, mechanically loaded fibrocartilaginous organ with limited regenerative potential [4].

Implant-associated infection and osteomyelitis further complicate orthopedic reconstruction. Titanium and titanium alloy implants provide excellent mechanical and corrosion properties, but bacterial adhesion and biofilm formation can occur before stable osseointegration is achieved. Once biofilm develops, bacteria become less accessible to systemic antibiotics and host immune mechanisms [5]. Therefore, orthopedic biomaterials increasingly need to combine regenerative, mechanical, anti-infective, delivery, and interface-stabilizing functions instead of simply filling space.

These diverse clinical problems require biomaterials that are biocompatible yet also structurally tunable, mechanically appropriate, biologically active, degradable when needed, antimicrobial when indicated, processable into multiple geometries, and compatible with local delivery of antibiotics, growth factors, cells, platelet products, nucleic acids, exosomes, or small molecules. Chitosan is attractive because it can be processed into multiple orthopedic formats and chemically modified for tissue-specific functions.

### 1.2. Chitosan as a Tunable Polymer Platform

Chitosan is a natural, chitin-derived, partially deacetylated aminopolysaccharide composed mainly of β-(1→4)-linked D-glucosamine and N-acetyl-D-glucosamine units. It is generally obtained by deacetylation of chitin, most commonly from crustacean shells, although fungal and insect sources are increasingly relevant. Historically, chitin was identified in the nineteenth century before chitosan was recognized as its deacetylated, acid-soluble derivative; Braconnot, Odier, Rouget, and Hoppe-Seyler are historically important because they established the chemical lineage from chitin to chitosan, while later polymer-science sources clarified that biomedical chitosan cannot be treated as a chemically uniform material [6,7,8,9,10,11,12]. Modern chitosan describes a family of related polymers whose molecular weight, degree of deacetylation, acetylation pattern, crystallinity, viscosity, solubility, impurity profile, source, and processing history can vary substantially [13,14,15,16].

The defining chemical feature of chitosan is the primary amino groups on its glucosamine units, which protonate under acidic conditions to give a pH-dependent cationic charge. This cationic character distinguishes chitosan from many polysaccharides and underlies several orthopedically relevant functions: electrostatic interaction with negatively charged matrix components (glycosaminoglycans, hyaluronic acid, and chondroitin sulfate), proteins, DNA, bacterial membranes, and blood components; polyelectrolyte complexation; hemostatic behavior; mucoadhesion; antimicrobial activity; nanoparticle formation; and growth-factor or drug binding [12,17,18].

For orthopedic biomaterials, the most important chitosan variables are degree of deacetylation, molecular weight, viscosity, charge density, solubility, degradation rate, sterilization sensitivity, residual impurities, and derivative chemistry. Degree of deacetylation affects charge density, hydrophilicity, crystallinity, degradation, cell response, antimicrobial behavior, and protein interaction, while molecular weight influences viscosity, injectability, mechanical reinforcement, degradation, and release kinetics. These variables determine whether chitosan behaves as a soluble polymer, injectable hydrogel precursor, scaffold matrix, antimicrobial coating, nanoparticle carrier, bioadhesive, or cement additive [19,20,21].

Chitosan source and processing are also biologically relevant: biological source, extraction, deacetylation, purification, and nanomaterial processing influence chitosan structure, molecular weight, acetylation profile, solubility, and application suitability [22,23,24,25,26,27]. Derivative nanoparticles further expand delivery, stability, and biomedical performance through chemical modification, supporting the view that “chitosan-based” systems should be interpreted by formulation rather than polymer name alone [28].

Chitosan can be processed into porous freeze-dried scaffolds, membranes, films, fibers, electrospun mats, microspheres, nanoparticles, injectable thermosensitive and photocrosslinkable hydrogels, implant coatings, bioadhesives, and bone-cement additives, and chemically modified into carboxymethyl, quaternized, sulfated, glycol, methacrylated, catechol-modified, and other derivatives. In orthopedic applications, it should therefore be understood not as a single fixed biomaterial but as a tunable polymer platform whose performance depends on source, chemistry, architecture, processing, and target tissue.

### 1.3. Orthopedic Application Landscape and Translational Gap

The orthopedic relevance of chitosan arises from the match between its polymer chemistry and the functional demands of musculoskeletal tissues. In cartilage and osteochondral repair, chitosan can support matrix retention, hydrogel formation, scaffold architecture, and blood-clot stabilization after marrow stimulation. In bone regeneration, chitosan is commonly combined with hydroxyapatite (HAp), nano-hydroxyapatite (nano-HAp), calcium phosphate, bioactive glass, collagen, gelatin, silk fibroin, or other reinforcement phases to create osteoconductive and osteoinductive composite scaffolds. In implant infection and osteomyelitis, chitosan can act as an antimicrobial polymer, local antibiotic carrier, silver-release modulator, quaternized contact-killing interface, or injectable cavity-filling hydrogel. In bone cement, chitosan can modify injectability, setting behavior, washout resistance, bioactivity, antibacterial behavior, and mechanical properties. In tendon, ligament, enthesis, and rotator cuff repair, chitosan can be processed into aligned fibers, anti-adhesion barriers, PRP-retentive hydrogels, immunomodulatory interfaces, and catechol-modified bioadhesives. In intervertebral disk repair, chitosan’s cationic structure can support proteoglycan retention, nucleus pulposus-like matrix formation, annulus fibrosus repair, and injectable hydrogel delivery.

The breadth of these applications creates a major interpretive challenge: a chitosan bone scaffold, a chitosan–blood cartilage implant, a quaternized titanium coating, a chitosan-modified PMMA cement, a catechol tendon bioadhesive, and a chitosan disk hydrogel are not equivalent materials, differing in chemistry, architecture, mechanical requirements, degradation, payload strategy, biological target, surgical workflow, and translational pathway. Orthopedic chitosan evidence therefore cannot be interpreted by asking whether “chitosan works” in general, but by asking which formulation works, for which indication, through which mechanism, under which mechanical environment, and against which clinically relevant alternative.

Existing reviews have addressed chitosan in bone tissue engineering, cartilage repair, mineralized tissues, osteoarthritis delivery, dental/craniofacial applications, and general biomedical delivery. Bone-focused reviews have covered chitosan scaffolds, mineralized-tissue regeneration, and bone-disorder treatment across rigid scaffolds, hydrogels, membranes, microspheres, nanoparticles, and delivery systems [29,30,31,32,33,34,35]. Cartilage- and osteoarthritis-focused reviews have emphasized chondrogenic scaffolds, knee cartilage regeneration, viscosupplementation, intra-articular residence, drug delivery, and anti-inflammatory/antioxidant strategies [36,37,38]. Growth-factor-focused synthesis has highlighted functionalization of chitosan bone scaffolds, particularly controlled release and osteogenesis–angiogenesis coupling [39].

Additional review-level sources further support the need for cross-domain orthopedic synthesis. Rodríguez-Vázquez et al. reviewed chitosan as a scaffold material for regenerative medicine, Kozusko et al. focused specifically on chitosan as a bone scaffold biomaterial, and Kim et al. summarized chitosan-based biomaterials for broader tissue-regeneration applications [40,41,42]. These reviews reinforce the rationale for interpreting chitosan through polymer design, scaffold architecture, and application-specific performance, not as a single uniform material.

Early orthopedic synthesis by Di Martino et al. framed chitosan as a versatile biopolymer for cartilage, intervertebral disk, and bone tissue engineering because of its low foreign-body reaction, antibacterial behavior, moldability into porous structures, controlled-delivery capacity, and cationic DNA-complexing potential [43]. Since then, orthopedic chitosan research has expanded from simple porous scaffolds to mineralized composites, chitosan–blood cartilage implants, injectable hydrogels, growth-factor microspheres, antibiotic-eluting and quaternized anti-biofilm coatings, bone-cement modifiers, tendon-to-bone bioadhesives, PRP-loaded rotator cuff hydrogels, and disk hydrogels. However, this literature remains distributed across separate tissue- and material-specific domains, so a combined synthesis is needed to connect polymer structure, format, quantitative performance, biological mechanism, preclinical model, clinical evidence, and translational maturity within a single structure–property–performance framework.

### 1.4. Aim and Scope of This Structured Narrative Review

To aid navigation of this integrated, multi-domain review, the overall structure is summarized below; the review is organized as a single cross-domain synthesis rather than as separate manuscripts so that polymer chemistry, material format, quantitative performance, and translational maturity can be compared on a common framework.

Manuscript roadmap.

**Section**

**Focus**

Section 2
Review methodology, evidence-based development, and evidence-quality/risk-of-bias boundaries
Section 3
Polymer identity, derivative chemistry, and the structure–property–performance logic, including the core property–performance table
Section 4
Application-specific synthesis across preclinical and clinical cartilage, bone regeneration, cross-cutting delivery, anti-infective interfaces, bone cements, tendon/rotator cuff, and intervertebral disk domains
Section 5
Critical interpretation, clinical-readiness summary, negative and neutral evidence, evidence limitations, and perspectives
Section 6
Indication-specific claim boundaries and conclusions


The aim of this structured narrative review is to synthesize the final evidence base of 258 unique publications on the orthopedic use of chitosan and its polymer-science context, covering chitosan-based scaffolds, hydrogels, coatings, nanoparticles, microspheres, fibers, bioadhesives, bone cements, cartilage repair adjuncts, tendon-to-bone and rotator cuff systems, anti-infective systems, and disk biomaterials.

This review provides a structured, domain-based synthesis of orthopedic chitosan biomaterials, with emphasis on polymer design, quantitative performance, and translational maturity instead of pooled effect estimation. The descriptive quantitative component refers to the extraction and interpretation of numerical material, biological, preclinical, biomechanical, imaging, antibacterial, release-kinetic, and clinical endpoints within a narrative-review framework.

The review is organized around four linked objectives: first, to identify the main orthopedic formats in which chitosan has been investigated (scaffolds, hydrogels, coatings, cements, fibers, delivery systems, and bioadhesives); second, to summarize key quantitative endpoints across domains, from polymer variables (molecular weight, degree of deacetylation) and material/mechanical properties to antibacterial, biological, imaging, patient-reported, and biomechanical outcomes; third, to compare translational maturity across indications, distinguishing clinically tested cartilage repair from predominantly preclinical bone, infection, cement, tendon, rotator cuff, and disk applications; and fourth, to identify reporting gaps, negative or neutral evidence, and priority studies for clinical translation.

Rather than treating chitosan as a single material, this review evaluates it as a tunable orthopedic polymer platform. This structure is intended to provide a critical synthesis for readers in polymer science, biomaterials, and orthopedic translational research.

## 2. Review Methodology, Evidence Base, and Narrative Synthesis Framework

This section describes the literature identification, selection, extraction, evidence-base development, and narrative synthesis approach used in this structured narrative review with descriptive quantitative endpoint extraction. The methods were designed to support transparent evidence-base development, domain-level organization, formulation-specific interpretation, endpoint extraction, evidence weighting, and translational maturity comparison across heterogeneous orthopedic chitosan applications.

### 2.1. Review Design

This article was designed as a structured narrative review with descriptive quantitative endpoint extraction, evaluating orthopedic chitosan-based biomaterials across regenerative, anti-infective, mechanical-interface, and clinical–translational applications. Because the literature is highly heterogeneous in chemistry, derivative type, architecture, formulation, anatomical target, model, defect size, comparator, outcome, and follow-up, statistical pooling and meta-analysis were not appropriate.

The methodology was based on a final evidence base of 258 unique publications, emphasizing descriptive extraction of quantitative endpoints, domain-level synthesis, formulation-specific interpretation, and translational maturity assessment. The descriptive quantitative component refers to the extraction, tabulation, and interpretation of numerical material, biological, preclinical, biomechanical, imaging, antibacterial, release-kinetic, and clinical endpoints within a narrative framework.

Orthopedic chitosan research spans multiple non-equivalent domains—cartilage repair, bone regeneration, implant coatings, osteomyelitis, bone cement, tendon/ligament repair, rotator cuff tendon-to-bone healing, and disk regeneration—that cannot be meaningfully pooled but can be compared narratively using shared polymer-science and translational variables (chemistry, format, porosity, pore size, mechanics, gelation, degradation, release kinetics, antibacterial efficacy, histology, imaging, biomechanics, clinical follow-up, and maturity).

All identification, screening, evidence-base development, domain assignment, and extraction were performed by the author in two sequential passes to improve consistency and reduce transcription error: the first identified candidates, assigned preliminary domains, and extracted available endpoints; the second rechecked relevance, duplicate status, DOI-to-PMID reconciliation, domain assignment, and extracted numerical data against the available sources.

The review addressed three linked questions: (i) Which chitosan-based formats have been investigated for orthopedic or orthopedic-adjacent applications? (ii) Which quantitative material, biological, preclinical, biomechanical, imaging, antibacterial, release-kinetic, or clinical outcomes support their use? (iii) Which indications have reached clinical or near-clinical maturity? This approach was selected because prior reviews have generally focused on isolated areas such as bone tissue engineering, cartilage repair, mineralized tissues, or general tissue regeneration, whereas the present review integrates orthopedic chitosan evidence across multiple application domains within a single structure–property–performance framework.

### 2.2. Data Sources and Literature Identification Strategy

Literature identification, screening, evidence-base development, and quantitative extraction were conducted between 1 January 2026 and 1 May 2026. PubMed/MEDLINE was the primary structured database, supplemented by targeted Web of Science, Scopus, and Google Scholar searches for citation-linked, non-PubMed-indexed, online-first, DOI-only, and review/polymer-science sources; PubMed Central, Europe PMC, publisher full texts, DOI-linked pages, and backward/forward citation tracking were used to verify source identity and retrieve full-text or metadata when available.

No formal publication-date restriction was applied at the search stage because the review aimed to capture both foundational chitosan polymer-science sources and orthopedic application studies. The final evidence base therefore included foundational polymer-science sources from 1986 onward and orthopedic application studies from 1999 onward. No automated language filter was applied during the initial search process; however, extraction and interpretation were limited to publications for which sufficient English-language title, abstract, full-text, PubMed metadata, DOI-linked publisher information, or indexed bibliographic information was available. Publications without sufficient methodological, material, clinical, or quantitative detail were not used to support strong efficacy claims.

The search strategy used four conceptual blocks—material (chitosan and its derivatives), orthopedic tissues and indications, biomaterial formats, and translation/study type—combined with Boolean operators; the full term list for each block is provided in Supplementary Table S6 (sheet S6C).

Representative PubMed/MEDLINE search strings included the following.

(chitosan OR chitosan-based OR carboxymethyl chitosan OR quaternized chitosan OR sulfated chitosan OR glycol chitosan) AND (orthopedic OR bone OR cartilage OR osteochondral OR tendon OR ligament OR rotator cuff OR intervertebral disk OR titanium OR osteomyelitis OR bone cement)

(chitosan OR BST-CarGel OR chitosan-glycerol phosphate OR chitosan blood implant) AND (microfracture OR bone marrow stimulation OR cartilage repair OR osteochondral lesion)

(chitosan OR quaternized chitosan OR carboxymethyl chitosan) AND (titanium OR implant coating OR biofilm OR osteomyelitis OR vancomycin OR gentamicin)

(chitosan OR chitosan microspheres OR chitosan nanoparticles) AND (BMP-2 OR VEGF OR TGF-beta OR platelet lysate OR PRP OR alendronate OR growth factor) AND bone regeneration

(chitosan OR chitosan-glycerophosphate OR chitosan hydrogel) AND (nucleus pulposus OR annulus fibrosus OR intervertebral disk OR disk degeneration)

Equivalent keyword combinations were adapted for Web of Science, Scopus, and Google Scholar and used mainly for citation chasing, DOI verification, identification of non-PubMed-indexed polymer-science and online-first materials-science articles, and confirmation that key orthopedic or translational records had not been missed by PubMed/MEDLINE alone.

All included publications were assessed at the full-text level.

Backward and forward citation tracking from high-yield articles—especially the multicenter BST-CarGel randomized controlled trial, its 5-year follow-up, the osteochondral biopsy substudy, and systematic reviews of chitosan/nano-HAp and growth-factor-functionalized scaffolds—was used to identify clinically important studies, older polymer-science sources, non-PubMed-indexed articles, and application-specific studies not captured by a single database query.

**Figure 1:**
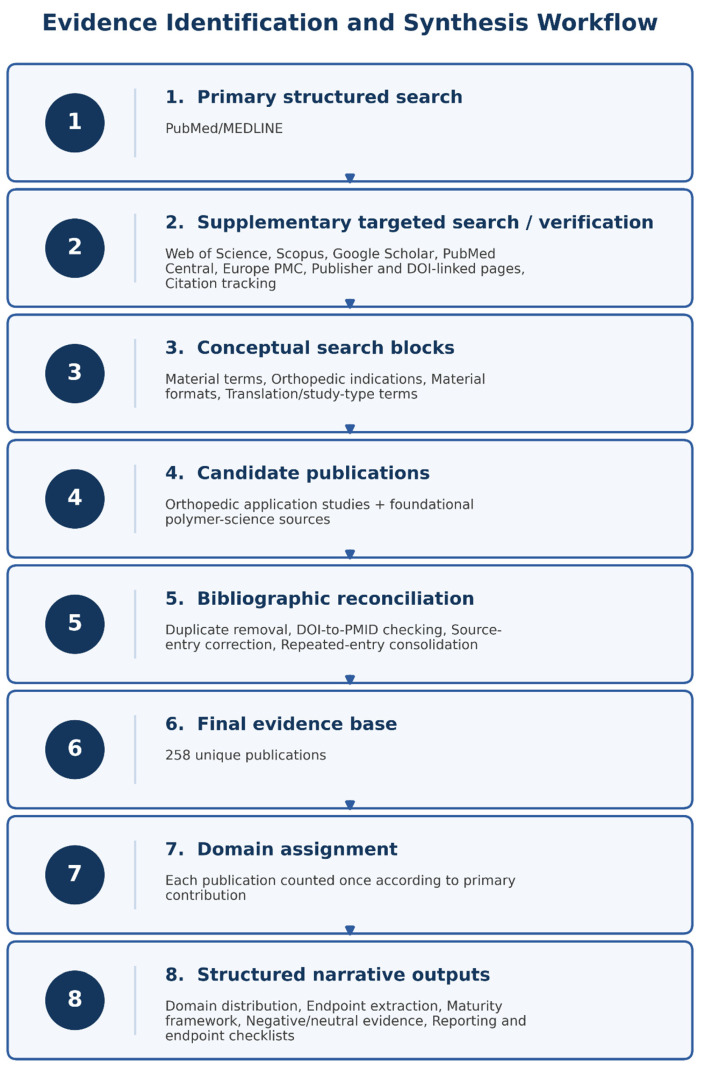
Descriptive evidence-base development process for the structured narrative review. PubMed/MEDLINE was used as the primary structured search database. Web of Science, Scopus, and Google Scholar were used as supplementary targeted search and verification tools to identify citation-linked publications, non-PubMed-indexed materials-science articles, recent online-first sources, and DOI-only records. PubMed Central, Europe PMC, publisher-hosted full texts, DOI-linked pages, and backward/forward citation tracking were also used to retrieve or verify source information. Candidate publications were reconciled through duplicate removal, DOI-to-PMID checking, correction of source-entry inconsistencies, and consolidation of repeated publication entries. The final evidence base included 258 unique publications, each counted once according to its primary contribution. This figure maps the evidence-base development and synthesis workflow used for the structured narrative review, including source identification, supplementary verification, bibliographic reconciliation, domain assignment, and structured evidence synthesis.

Web of Science, Scopus, and Google Scholar were used as supplementary targeted search and verification sources rather than as independently reported systematic databases. The evidence base is intended to be illustrative and interpretive rather than an exhaustive database-level capture of all orthopedic chitosan publications. Database-specific hit counts, duplicate counts, title/abstract screening counts, full-text exclusion counts, and exclusion reasons were not retained for every supplementary source. Therefore, this review does not claim PRISMA-ScR compliance, and the workflow in Figure 1 should be interpreted as a descriptive evidence-development map rather than a systematic-review flow diagram. A consolidated summary of the databases and verification sources, the four conceptual blocks and their search terms, the representative PubMed/MEDLINE strings, and the search parameters is provided in Supplementary Table S6.

### 2.3. Literature Selection Framework

Publications were eligible when they met at least one criterion: (i) chitosan, chitin, or a modified derivative used as a core structural, functional, delivery, coating, adhesive, antimicrobial, or cement-modifying component; (ii) an orthopedic or musculoskeletal application (bone, cartilage, tendon, ligament, enthesis, spine, implant, osteomyelitis, or bone cement); (iii) directly relevant mineralized-tissue or polymer-science information needed to interpret orthopedic chitosan; or (iv) material, biological, preclinical, clinical, technical, economic, or review-level information relevant to orthopedic biomaterial design or translation.

Included publication types were intentionally broad because the review aimed to synthesize a heterogeneous translational field rather than to pool one narrowly defined clinical endpoint. Eligible publications included narrative reviews, systematic reviews, polymer-science reviews, in vitro biomaterial studies, cell-culture studies, animal studies, large-animal translational models, human cadaveric or ex vivo biomechanical studies, surgical technique papers, clinical case series, comparative clinical studies, randomized controlled trials, health-economic evaluations, and mechanistic studies.

Primary application studies were prioritized for domain-specific synthesis. Review-level publications were used mainly to support background, contextual interpretation, and identification of broader evidence patterns. They were not treated as equivalent to primary experimental or clinical studies when assigning translational maturity.

Publications were excluded from the core synthesis if chitosan was only a minor laboratory reagent, if the application was unrelated to musculoskeletal tissue or orthopedic translation, if it addressed general non-musculoskeletal wound healing without a clear orthopedic link, or if it lacked sufficient material, biological, clinical, or translational information for meaningful interpretation.

Dental, periodontal, and craniofacial studies were handled conservatively: excluded when purely dental/periodontal without broader mineralized-tissue relevance, but retained when they provided direct information on bone regeneration, chitosan-modified grafts, hydroxyapatite/calcium phosphate composites, osteogenic differentiation, guided bone-regeneration membranes, graft bioactivity, or mineralized-tissue scaffold design. Such studies informed polymer–bone biology but were not treated as direct evidence of efficacy in load-bearing defects unless the model or endpoint supported it.

The operational rules applied to decide inclusion and interpretation across source categories are summarized in Table 1.

These rules were applied consistently across both author-led screening passes; borderline records were resolved in favor of the more conservative interpretation.

### 2.4. Evidence-Base Development and Deduplication

The final evidence base comprised 258 unique chitosan-related publications spanning 1986–2026 (Supplementary Table S1); a small number of additional contextual and methodological references (e.g., general orthopedic background sources and the narrative-review appraisal tool) are cited separately and are not part of this evidence base (the reference list comprises 265 entries in total, whereas the evidence base itself comprises 258 unique publications), with orthopedic application studies beginning in 1999.

Duplicate handling was performed at three levels: exact duplicates were removed; repeated clinical, technical, or experimental publications were reconciled using PubMed ID, DOI, title, first author, year, and study identity; and DOI-only sources were retained when no PubMed ID was available (classic polymer-science reviews, historical sources, recent non-PubMed-indexed articles). Publications spanning more than one theme were counted once and assigned to the most specific primary domain.

The domain-level distribution is summarized in Section 4.1 and the complete study-level table in Supplementary Table S1. By study type, the evidence base comprised approximately 198 primary preclinical studies (in vitro and/or animal; ~76.7%), 21 human clinical studies (~8.1%), 38 reviews (including 4 systematic reviews; ~14.7%), and a few polymer-science/background sources (~0.4%); the evidence is therefore predominantly preclinical, and not all 258 publications are primary orthopedic application studies. The final evidence base of 258 unique publications is fully listed in Supplementary Table S1, with each publication represented in the text as an individually discussed study or part of a clustered thematic citation.

The evidence-base development process is summarized descriptively in Figure 1.

### 2.5. Data Extraction Variables

Data extraction supported structured narrative synthesis and domain-level interpretation rather than risk-of-bias-weighted effect estimation. For each publication, bibliographic identifiers, study type, domain, and target tissue, chitosan chemistry and format, fabrication, key material/mechanical/release/antibacterial/biological/imaging/histological/marker/clinical endpoints, model, and comparator were extracted whenever available; the full variable set and extracted data are provided in Supplementary Tables S1 and S2.

Because chitosan’s performance is formulation-dependent, special attention was given to chitosan chemistry and architecture. Extracted derivative types included native chitosan, carboxymethyl chitosan, quaternized chitosan/HACC, sulfated chitosan, glycol chitosan, methacrylated chitosan, and catechol-modified chitosan. Extracted formats included porous scaffolds, freeze-dried scaffolds, electrospun fibers, hydrogels, injectable thermosensitive hydrogels, nanoparticles, microspheres, coatings, membranes, bioadhesives, bone cement additives, and 3D-printed constructs.

Quantitative extraction was emphasized throughout. For example, visible-light photocrosslinked methacrylated glycol chitosan/hyaluronic acid hydrogels gave stable gels with 87–90% chondrocyte viability after 40 s irradiation, with longer irradiation increasing modulus but reducing viability [44]; bacterial nanocellulose–chitosan–gelatin–hydroxyapatite scaffolds had pore diameters of 384.5–457.4 µm (surface) and 467.5–498.7 µm (interior) with 66.0–81.4% porosity [45]; and calcium phosphate cement–chitosan systems improved setting time and flexural strength [46].

### 2.6. Domain-Specific Outcome Extraction

Outcome extraction was adapted to each orthopedic domain because relevant endpoints differ substantially across tissues.

For cartilage and osteochondral repair, extracted outcomes included histological matrix markers (e.g., glycosaminoglycan, collagen II, and SOX9), scaffold integration, ICRS/MOCART scoring, T2 relaxation, lesion filling, and patient-reported scores, with the full endpoint set in Supplementary Table S5. The BST-CarGel randomized controlled trial provided key quantitative MRI endpoints: lesion filling 92.8 ± 2.0% versus 85.2 ± 2.1% and T2 relaxation 70.5 ± 4.5 ms versus 85.0 ± 4.9 ms at 12 months for BST-CarGel plus microfracture versus microfracture alone [47].

For bone regeneration, extracted outcomes included osteogenic markers, micro-CT bone volume and BV/TV, trabecular microarchitecture, histomorphometry, mechanical repair strength, and immune-cell polarization, with the full endpoint set in Supplementary Table S5. For example, ultralong hydroxyapatite microtube/chitosan scaffolds were evaluated using rat critical-size calvarial defect outcomes, including BV/TV 14.07 ± 0.84% at 60 days versus 9.74 ± 1.36% for chitosan alone [48].

For implant coatings and infection control, extracted outcomes included antibacterial endpoints, release duration, corrosion, osteoblast compatibility, osseointegration, and mechanical integrity, with the full endpoint set in Supplementary Table S5. Chitosan-containing coatings were evaluated in contexts ranging from antimicrobial titanium coatings to silver/chitosan hybrid coatings, vancomycin-loaded systems, and screw-level chemical immobilization [49,50,51].

For bone cement, extracted outcomes included handling, mechanical, and ISO-relevant safety endpoints, with elution kinetics, biofilm inhibition, and osteoblast adhesion detailed in Supplementary Table S5. Negative evidence was intentionally retained, especially where chitosan improved biological behavior but compromised mechanical performance or antibiotic release [52].

For tendon, ligament, enthesis and rotator cuff repair, extracted outcomes included tenogenic markers, tendon-to-bone histology, fibrocartilage, vascularization, macrophage phenotype, and biomechanics (load-to-failure, stiffness, pull-out, failure mode), with the full endpoint set in Supplementary Table S5. Chitosan–gelatin–glycerol phosphate hydrogel as a collagenase carrier, for example, improved rabbit tendon–bone healing with load-to-failure 23.8 ± 8.13 N versus 14.3 ± 3.9 N in controls [53].

For intervertebral disk applications, extracted outcomes included gelation, injectability, NP matrix markers, compressive modulus, disk height/DHI, MRI T2, extrusion resistance, annulus repair, and large-animal feasibility, with the full endpoint set in Supplementary Table S5. Triple-interpenetrating dextran/chitosan/teleostean hydrogel studies were interpreted as advanced translational disk evidence because they included cadaveric biomechanical testing, 10,000 loading cycles, and goat-model translation [54,55].

### 2.7. Author-Defined Translational Maturity Framework

An author-defined translational maturity framework was used to compare orthopedic chitosan applications across domains. This framework was not intended to replace formal regulatory technology-readiness levels, clinical guideline grading systems, or validated risk-of-bias instruments. It was used as a narrative-review tool to distinguish early material characterization, in vitro testing, animal proof-of-concept, large-animal validation, human technical feasibility, observational clinical evidence, comparative clinical evidence, randomized or long-term clinical evidence, and routine clinical adoption. Maturity levels were assigned by the sole author during the two-pass review process and were not independently rated or externally validated. They should therefore be interpreted as an exploratory cross-domain heuristic rather than as formal evidence grades.

The proposed maturity framework was as follows:

Level 1: Polymer synthesis, physicochemical characterization, or material fabrication only.

Level 2: In vitro cell, antibacterial, release-kinetic, or material-performance testing.

Level 3: Small-animal in vivo proof-of-concept.

Level 4: Large-animal in vivo proof-of-concept.

Level 5: Human technical feasibility, cadaveric testing, or surgical workflow demonstration.

Level 6: Human observational clinical evidence, including case series or cohort studies.

Level 7: Comparative clinical evidence.

Level 8: Randomized clinical trial, biopsy-level clinical evidence, or long-term clinical follow-up.

Level 9: Routine clinical adoption, registry-level durability, reimbursement-level evidence, or guideline-level integration.

Using this framework, knee cartilage repair with BST-CarGel/chitosan–blood implant reached the highest maturity among orthopedic chitosan applications because it included a multicenter randomized controlled trial, 5-year follow-up, and a human osteochondral biopsy substudy [47,56,57]. Talus, hip, and patellar cartilage repair were classified as intermediate maturity because they included technical papers, observational cohorts, comparative clinical studies, MRI follow-up, or mid-term follow-up, but lacked the same level of randomized long-term evidence as knee cartilage repair.

Bone regeneration, implant coatings, osteomyelitis hydrogels, bone cements, tendon-to-bone systems, rotator cuff hydrogels, and disk biomaterials were generally classified as preclinical or translational-preclinical, depending on whether evidence was limited to in vitro, small-animal, large-animal, cadaveric, or hardware-level validation. Maturity was interpreted together with study type, anatomical indication, comparator quality, follow-up duration, mechanical relevance, and endpoint applicability.

Because evidence within a domain was sometimes distributed across adjacent tiers, maturity was recorded as a single level when a domain’s qualifying evidence mapped clearly to one tier and as a level range (for example, Levels 6–7 or Levels 2–4) when the qualifying studies straddled two or more adjacent tiers, either because the strongest individual study and the breadth of supporting evidence indicated different tiers or because heterogeneous designs such as in vitro, small-animal, and large-animal evidence coexisted within the same domain. Ranges were reported conservatively rather than reduced to a single point, with the lower bound reflecting the most consistently supported tier. To check the stability of the resulting cross-domain ordering, each range was collapsed to both its lower and its upper bound; under both bounds the qualitative ranking was preserved, with clinically tested knee cartilage repair remaining the most mature domain, the other cartilage indications and the intervertebral disk/spine domain occupying intermediate positions, and bone regeneration, implant coatings, osteomyelitis hydrogels, bone cements, and tendon/rotator cuff systems remaining predominantly preclinical. The ordering was therefore stable to one-level shifts at boundary cases, although the absolute level assigned to any single domain may vary within its stated range.

For visual evidence mapping, the nine-level maturity framework was additionally summarized into application-by-evidence-category intensity scores from 0 to 3. These figure-level scores indicate the relative presence and strength of evidence within each evidence category for a given application domain: 0 = absent or minimal evidence, 1 = limited evidence, 2 = moderate evidence, and 3 = strongest evidence level observed in this review. These scores were used only to visualize cross-domain evidence distribution and should not be interpreted as formal certainty grades, risk-of-bias scores, or regulatory readiness levels. Each 0–3 score was assigned according to the best available study design, anatomical and mechanical relevance of the model, and consistency of findings within that evidence category rather than publication count alone; the application-level rationale and representative anchors are detailed in Supplementary Table S2, sheet S2K.

### 2.8. Handling of Conflicting and Negative Evidence

Conflicting and negative evidence was deliberately retained. This was essential because chitosan performance is formulation-dependent, and overgeneralization would misrepresent the field. Several studies showed that chitosan-containing systems can fail to outperform comparators or may introduce trade-offs. A composite chitosan-reinforced osteochondral scaffold failed to provide superior osteochondral regeneration in preclinical models [58]. A rapidly degrading 10 kDa chitosan–blood implant did not improve cartilage resurfacing compared with blood implant controls in a sheep model, illustrating that favorable rabbit results may not translate directly to larger animals and different surgical geometries [59]. Chitosan incorporation into gentamicin-loaded Palacos R acrylic cement decreased gentamicin release, failed to reduce biofilm formation, and impaired mechanical compliance [52]. In critical-size radial bone defects, chitosan alone did not promote considerable new bone formation, whereas gelatin and gelatin–chitosan performed better [60].

These studies were used to support balanced interpretation and to identify formulation-level limitations, including insufficient mechanical strength, inadequate degradation behavior, poor integration, reduced antibiotic elution, species-specific healing differences, and inadequate scaffold architecture.

### 2.9. Evidence Weighting, Descriptive Quantitative Synthesis, and Methodological Limitations

Given the heterogeneity in chitosan chemistry, derivative type, format, indication, model, comparator, endpoint, and follow-up, statistical pooling and meta-analysis were not performed; the quantitative component refers to extraction, tabulation, and narrative interpretation of reported endpoints rather than pooled effect-size estimation. Priority was given to endpoints informing orthopedic material design or translation, such as porosity, pore size, compressive/tensile and adhesive properties, gelation, degradation, release duration, antibacterial activity, cell viability, osteogenic/chondrogenic markers, bone volume, cartilage MRI parameters, patient-reported outcomes, and failure load.

Evidence was weighted narratively according to study design, comparator quality, anatomical relevance, mechanical relevance, endpoint type, follow-up duration, and translational maturity. Randomized clinical evidence, long-term follow-up, and biopsy-level human data were interpreted as stronger than uncontrolled observational series or surgical technique reports. For preclinical applications, large-animal studies, cadaveric biomechanical testing, clinically relevant defect models, hardware-level validation, and simultaneous mechanical-biological testing were interpreted as more translationally informative than isolated cell-culture or material-characterization studies. In vitro antibacterial activity was not interpreted as sufficient evidence of anti-infective efficacy unless supported by cytocompatibility, release-kinetic, osseointegration, mechanical, or in vivo infection data.

This review was not conducted as a PRISMA-ScR scoping review, formal systematic review, or meta-analysis. To provide a transparent narrative-review quality self-check, the article was self-assessed using the Scale for the Assessment of Narrative Review Articles (SANRA), which rates six items from 0 to 2 (maximum 12). The author-assigned scores were: importance for the readership, 2; concrete aims, 2; description of the literature search, 1; referencing, 2; scientific reasoning, 2; and appropriate presentation of data, 2 (total, 11/12). The literature search item was deliberately scored 1 because the search, although described in terms of databases, conceptual blocks, representative strings, and time window, was interpretive rather than exhaustive, was performed by a single author, and did not retain database-specific hit, duplicate, or exclusion counts. This self-assessment is provided for transparency and is itself limited by being performed by the sole author [61]. No formal risk-of-bias tool, certainty-of-evidence grading system, or pooled quantitative model was applied. Literature identification, screening, domain assignment, translational maturity assignment, and quantitative extraction were performed by a single author in two sequential author-led passes. This approach improved internal consistency and reduced transcription error, but it does not replace independent dual-reviewer screening, independent extraction, or external adjudication. In addition, the use of PubMed/MEDLINE as the primary database and the restriction to records with sufficient English-language information introduce language and indexing selection bias; the non-English chitosan literature is therefore likely under-represented. Therefore, the translational maturity framework should be interpreted as an author-defined exploratory heuristic rather than as a formal evidence grade.

Negative and neutral studies were deliberately retained because they define formulation boundaries and reduce overgeneralization of positive chitosan findings.

### 2.10. Evidence-Quality and Risk-of-Bias Boundaries

Because this review compares translational maturity across heterogeneous domains, the boundaries of its evidence-quality appraisal are stated explicitly. No formal risk-of-bias or certainty-of-evidence instrument, such as RoB 2, ROBINS-I, SYRCLE, or GRADE, was applied. The translational maturity framework should therefore be interpreted as a translation-stage mapping heuristic rather than a certainty-of-evidence grade, and study type was deliberately not equated with evidence strength: a randomized trial does not by itself confer generalizability, and a domain assigned high maturity may still contain individual studies at meaningful risk of bias.

Within these limits, evidence was weighted narratively by endpoint type, comparator quality, anatomical and mechanical relevance, follow-up duration, and consistency of findings, rather than by publication count or design label alone. Preclinical in vivo studies were treated as heterogeneous in internal validity because randomization, allocation concealment, blinding, and sample-size justification were frequently unreported; clinical evidence was interpreted with attention to confounding, comparator choice, and outcome domain, as illustrated by the consistent structural but less consistent patient-reported signal for BST-CarGel. These boundaries should be kept in mind wherever maturity levels or intensity scores are cited in Section 4 and Section 5.

## 3. Chitosan as an Orthopedic Polymer Platform: Structure–Property–Performance Framework

This review interprets chitosan as a chemistry-defined orthopedic polymer platform, not a single biomaterial. Across the final evidence base of 258 unique publications, “chitosan-based” referred to highly different systems—soluble low-molecular-weight chitosan–blood implants, mineralized porous scaffolds, injectable hydrogels, quaternized coatings, nanoparticles, microspheres, fibers, catechol bioadhesives, calcium phosphate cement additives, PMMA modifiers, and disk hydrogels—that cannot be treated as equivalent simply because they contain chitosan.

**Figure 2:**
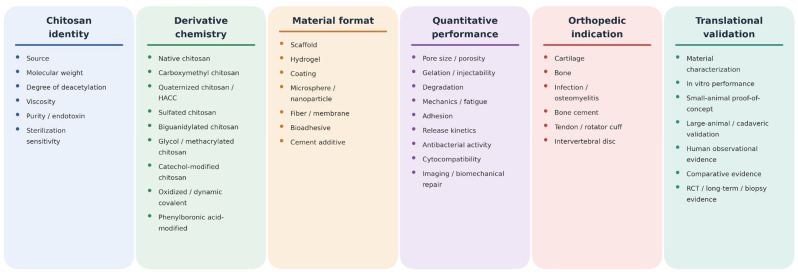
Structure–property–performance framework for orthopedic chitosan biomaterials. Chitosan performance depends on polymer identity, including source, molecular weight, degree of deacetylation, viscosity, purity, endotoxin status, and sterilization sensitivity. These variables interact with derivative chemistry, material format, composite phase, crosslinking strategy, payload, degradation behavior, and target-tissue mechanics. The same polymer family can therefore function as a scaffold, hydrogel, coating, particle carrier, fiber, membrane, bioadhesive, or cement additive, but each indication requires different quantitative endpoints and translational validation.

The structure–property–performance framework used throughout (Figure 2) connects polymer identity, derivative chemistry, material format, quantitative performance, orthopedic indication, and translational maturity, organizing the evidence around formulation-specific function rather than generic interpretation.

### 3.1. Polymer Identity and Derivative Chemistry

The defining chemical feature of chitosan is its glucosamine-rich backbone of primary amine groups, which protonate under acidic conditions to give a pH-dependent cationic charge. This charge explains many orthopedic functions: interaction with negatively charged matrix components (glycosaminoglycans, hyaluronic acid, and chondroitin sulfate), nucleic acids, proteins, bacterial membranes, blood components, and mineral surfaces; polyelectrolyte complexation; hemostatic and clot-stabilizing behavior; antimicrobial activity; nanoparticle formation; and local retention of drugs or biologics. These core property–performance relationships are summarized in Table 2.

Degree of deacetylation, molecular weight, viscosity, charge density, purity, solubility, crystallinity, degradation, and sterilization sensitivity are therefore primary design variables, not secondary descriptors: degree of deacetylation governs charge density, hydrophilicity, crystallinity, degradation, antimicrobial behavior, and cell response, while molecular weight governs viscosity, injectability, mechanical contribution, degradation rate, release kinetics, and immune response. A low-molecular-weight soluble chitosan–blood cartilage implant is therefore not equivalent to a high-molecular-weight fiber, quaternized coating, carboxymethyl hydrogel, or chitosan-modified PMMA cement. Vivcharenko et al. illustrate this: varying molecular weight and concentration substantially altered scaffold mechanics, pore diameter, and biological behavior, with a 2% medium-molecular-weight formulation (~153.9 kDa) identified as a promising cartilage-oriented composition [62].

Derivative chemistry should be selected by orthopedic function: carboxymethyl chitosan for aqueous solubility, injectability, and hydrogel formation; quaternized chitosan and HACC for antibacterial coatings, anti-biofilm interfaces, and antibacterial PMMA cement; sulfated chitosan for heparin-like growth-factor binding and osteogenesis–angiogenesis coupling; glycol and methacrylated derivatives for injectable or photocrosslinkable hydrogels; and catechol-modified chitosan for wet-tissue and tendon-to-bone adhesion. Emerging oxidized, biguanidylated, phenylboronic-acid-modified, and dynamic covalent derivatives further expand the design space toward antibacterial, self-healing, ROS-responsive, and immunomodulatory systems.

### 3.2. Material Format and Indication-Specific Performance Logic

Chitosan performance depends on material format. Porous scaffolds are most relevant to bone and some cartilage applications, where pore size, porosity, interconnectivity, wet compressive strength, mineral phase, degradation, and cellular infiltration are central. In bone regeneration, chitosan is rarely sufficient alone and is commonly reinforced with hydroxyapatite (HAp), nano-HAp, calcium phosphate, bioactive glass, collagen, gelatin, silk fibroin, or bacterial nanocellulose.

Injectable hydrogels are used in cartilage repair, osteomyelitis, tendon-to-bone repair, rotator cuff repair, and intervertebral disk regeneration. Their key variables include polymer concentration, pH, osmolality, gelation time, gelation temperature, injectability, swelling, degradation, modulus, adhesion, payload retention, and post-gelation cytocompatibility. Injectability alone is insufficient; the final gel must resist dilution, washout, extrusion, premature degradation, and mechanical failure.

Coatings are used mainly to functionalize titanium and titanium alloy implants, with key variables—adhesion, drug loading, release kinetics, antibacterial activity, corrosion resistance, osseointegration, and hardware mechanics—detailed in Supplementary Table S5. A coating that kills bacteria but impairs osteoblast function, promotes inflammatory bone remodeling, corrodes, or weakens hardware is not translationally adequate.

Microspheres and nanoparticles are delivery formats whose key variables—particle size, polydispersity, zeta potential, encapsulation efficiency, drug loading, release kinetics, retained bioactivity, and local pharmacokinetics—are detailed in Supplementary Table S5. Chitosan cationic charge supports complexation of proteins, platelet products, nucleic acids, antibiotics, and bisphosphonates; however, longer release is not automatically better and should match the biological window of the target indication.

Fibers, membranes, textiles, and bioadhesives are most relevant to tendon, ligament, annulus fibrosus, cartilage interfaces, and wet-tissue repair; their format-specific variables (alignment, fiber diameter, tensile strength, stiffness, and cyclic loading for tendon/ligament; wet adhesive strength, failure mode, handling time, and suture/anchor compatibility for bioadhesives) are detailed in Supplementary Table S5. Bone cement additives form a separate category: chitosan can improve injectability, setting, anti-washout behavior, bioactivity, antibacterial behavior, or elution, but can also compromise residual monomer, antibiotic release, bending strength, fatigue, or ISO-relevant safety.

### 3.3. Cross-Domain Design Rules and Transition to Evidence Synthesis

The evidence supports six cross-domain design rules. First, cationic charge is a shared mechanistic basis for antimicrobial behavior, clot stabilization, proteoglycan retention, nanoparticle formation, polyelectrolyte complexation, and interaction with negatively charged matrix components, but charge density must be balanced against cytotoxicity and inflammation. Second, architecture must match tissue function: mineralized porous constructs for bone, matrix-retentive hydrogels or scaffolds for cartilage, aligned or adhesive interfaces for tendon, injectable extrusion-resistant hydrogels for disk, antibacterial host-compatible interfaces for infection, and mechanically/aging-safe systems for cement.

Third, derivative chemistry should be function-driven, not interchangeable. Quaternization is useful for contact-killing and anti-biofilm behavior, carboxymethylation for solubility and injectable hydrogels, sulfation for growth-factor binding, methacrylation for photocrosslinking, and catechol modification for wet adhesion. Fourth, composite phase selection is decisive. HAp, nano-HAp, calcium phosphate, or bioactive glass are usually required for bone; hyaluronic acid, gelatin, collagen, chondroitin sulfate, GelMA, agarose, or decellularized cartilage matrix may support cartilage; and fibrous reinforcement or decellularized matrix may be needed for tendon and disk applications.

Fifth, biological testing and mechanical qualification must be simultaneous: a scaffold that raises osteogenic markers but collapses under load, a coating that kills bacteria but impairs osseointegration, a hydrogel that supports cells but extrudes, or a cement that improves bioactivity but fails ISO-relevant mechanics is not translationally mature. Sixth, negative and neutral findings define the design window—chitosan can improve cartilage, bone, infection, cement, tendon-to-bone, and disk repair, but can also fail when molecular weight, degradation, architecture, payload, comparator, or mechanical environment are poorly matched.

The following Results section applies this framework to the major orthopedic domains. Cartilage and osteochondral repair are analyzed separately as preclinical/mechanistic biomaterials and as clinical translation, because BST-CarGel/chitosan–blood implant has reached the highest available human evidence among orthopedic chitosan applications; bone regeneration as a broad preclinical domain of mineralized and functionalized composites; delivery systems as a cross-cutting carrier platform (growth factors, platelet products, antibiotics, ions, genes, microRNAs, small molecules, exosomes); and anti-infective interfaces, bone cements, tendon/rotator cuff systems, and disk applications according to application-specific endpoints and maturity.

## 4. Application-Specific Evidence Synthesis

### 4.1. Domain-Level Distribution and Translational Maturity of Orthopedic Chitosan Applications

The final evidence base included 258 unique publications after duplicate removal, DOI-to-PMID reconciliation, and consolidation of repeated entries, covering 1986–2026 (orthopedic application studies from 1999; foundational polymer-science sources from 1986). Sixteen publications appeared between 1999 and 2005, whereas 172 of 258 (66.7%) appeared between 2016 and 2026, indicating rapid expansion over the last decade, particularly in advanced hydrogels, smart coatings, osteoimmunomodulatory scaffolds, growth-factor delivery, microenvironment-responsive platforms, 3D-printed constructs, and clinical cartilage translation.

Each publication was assigned to one primary domain according to its dominant contribution; detailed study-level extraction is provided in Supplementary Table S1, while the main text retains a domain-level distribution (Table 3) for readability.

Bone scaffold/mineralized-tissue regeneration and cartilage/osteochondral biomaterials were the two largest application domains, together accounting for 96 of 258 publications, or 37.2% of the final evidence base. This distribution reflects the strong historical alignment between chitosan and extracellular-matrix-mimetic scaffold design. Foundational polymer-science and review-level sources represented 38 publications, or 14.7% of the final evidence base, and were retained because interpretation of orthopedic chitosan systems requires understanding of chitosan source, processing, molecular weight, degree of deacetylation, derivative chemistry, antimicrobial mechanism, characterization, and standardization.

**Figure 3:**
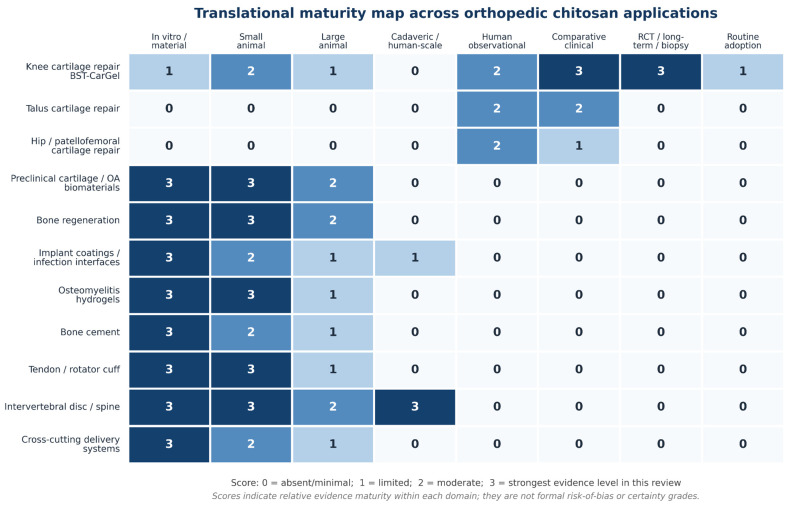
Translational maturity map of orthopedic chitosan applications. Knee cartilage repair with BST-CarGel/chitosan–blood implant had the strongest available human evidence, including randomized clinical evidence, 5-year structural follow-up, and biopsy-level data. Hip, talus, and patellar/patellofemoral cartilage repair had human clinical evidence but remained less definitive and joint-specific. Intervertebral disk/spine applications were among the most advanced non-cartilage preclinical-translational domains because they included cadaveric cyclic loading and large-animal delivery studies. Bone regeneration, implant coatings, osteomyelitis hydrogels, bone cements, tendon/rotator cuff systems, and cross-cutting delivery platforms remained predominantly preclinical or translational-preclinical. Scores are exploratory cross-domain maturity indicators and should not be interpreted as formal risk-of-bias scores, certainty-of-evidence grades, or regulatory readiness levels. Each 0–3 score reflects the best available study design, anatomical/mechanical relevance, and consistency of findings rather than publication count alone; the score rationale is provided in Supplementary Table S2, sheet S2K. These scores reflect a single author’s qualitative judgment and were not independently rated or externally validated.

Publication volume did not necessarily correspond to clinical maturity (Figure 3): bone regeneration was the largest cluster but remained predominantly preclinical, whereas knee cartilage repair had fewer publications but the strongest human evidence (randomized, 5-year imaging, and biopsy-level data). To separate volume from translational readiness, each major domain was summarized by its highest available evidence level and representative quantitative anchors (Table 4).

Intervertebral disk applications represented the most advanced non-cartilage preclinical-translational domain because they included cadaveric cyclic loading and goat-model delivery studies. Bone cement, coating, and infection-control studies provided some of the strongest quantitative material data, whereas tendon-to-bone and rotator cuff applications were mechanistically sophisticated but still largely preclinical.

Taken together, Table 3 and Table 4 show that orthopedic chitosan research is broad but unevenly mature: cartilage repair, especially BST-CarGel/chitosan–blood implant augmentation of knee microfracture, represents the clearest human clinical translation; bone regeneration is the broadest preclinical domain; and implant coatings, osteomyelitis hydrogels, bone cements, tendon-to-bone repair, rotator cuff applications, and disk biomaterials provide strong material and preclinical evidence but remain less clinically mature.

The remainder of the Results section synthesizes the evidence by orthopedic application domain instead of by publication chronology, addressing in turn preclinical cartilage and osteochondral biomaterials (4.2), clinical cartilage repair (4.3), bone regeneration (4.4), cross-cutting delivery systems (4.5), anti-infective interfaces and osteomyelitis systems (4.6), bone cements (4.7), tendon/ligament/enthesis/rotator cuff repair (4.8), and intervertebral disk and spine applications (4.9).

### 4.2. Cartilage and Osteochondral Repair: Preclinical Scaffold Systems, Hydrogel Design, and Chitosan–Blood Implant Mechanisms

Cartilage and osteochondral repair was one of the largest and most clinically relevant areas in the final evidence base: together, preclinical scaffold/hydrogel studies, osteoarthritis-oriented systems, and clinical cartilage publications accounted for 69 of 258 unique publications (26.7%). This section focuses on preclinical and mechanistic evidence (Section 4.3 covers clinical translation), interpreted through three design strategies: scaffold/hydrogel support for chondrogenesis, osteochondral or multiphasic repair, and chitosan–blood implant modulation of marrow stimulation.

#### 4.2.1. Scaffold and Hydrogel Design Principles

Early porous chitosan scaffold studies established feasibility but also revealed architectural constraints. Nettles et al. showed that freeze-dried chitosan scaffolds supported chondrocyte attachment, rounded morphology, proteoglycan deposition, and type II collagen production, but extracellular matrix accumulation was mainly peripheral because cell penetration into the scaffold interior was limited [91]. Earlier and related scaffold work using chitosan/chondroitin sulfate or chitosan/polyester systems supported the rationale for glycosaminoglycan-mimetic and hybrid natural–synthetic cartilage scaffolds [92,93]. Chitosan also functioned as a local delivery matrix: TGF-β1-loaded chitosan microspheres incorporated into porous chitosan scaffolds released TGF-β1 over approximately 5 days and increased chondrocyte proliferation and extracellular matrix production [94].

Natural-polymer combinations improved matrix formation and cell compatibility. Chitosan–gelatin scaffolds supported engineered elastic cartilage with a pore size of around 60–200 µm, seeding density of 50 × 10^6^ cells/mL, glycosaminoglycan content approaching 90%, and stiffness approaching 85% of native auricular tissue in a porcine model [95]. Chitosan physical hydrogels also acted as matrix-organizing “material decoys”, with chondrocytes aggregating and depositing cartilage-like matrix around hydrogel fragments at an optimal 1.5% *w*/*w* chitosan and 30–40% acetylation [96].

Injectable hydrogels addressed the geometric limitations of rigid scaffolds. Schiff-base chitosan–hyaluronic acid hydrogels supported chondrocyte survival without a toxic crosslinker, while chitosan–β-glycerophosphate–hydroxyethyl cellulose hydrogels gelled at 37 °C, supported MSC viability for 28 days, released insulin over 8 days, and supported TGF-β3-driven chondrogenesis [97,98]. Visible-light photocrosslinked methacrylated glycol chitosan/hyaluronic acid hydrogels showed a clear trade-off: 40 s irradiation gave stable gels with 87–90% viability, whereas 600 s raised compressive modulus to 11–17 kPa but reduced viability to 60–65% [44]. Carboxymethyl chitosan/oxidized chondroitin sulfate hydrogels with embedded microspheres combined injectability, compressive modulus around 13 kPa, and sustained protein release over 2 weeks [99].

Chitosan–hyaluronic acid systems showed that HA-containing matrices can support cartilage-like repair, but that adding cells or adjusting composition does not guarantee superior quality. Freeze-dried chitosan/HA scaffolds with 5% HA improved matrix production, while in rabbit osteochondral defects, chitosan–HA dialdehyde hydrogels produced repair tissue in all groups, with gel-only implants sometimes more native-like than cell-loaded gels [100,101]. Oxidized-maltodextrin-crosslinked chitosan/HA scaffolds reinforced the need to optimize stiffness, swelling, degradation, and chondrocyte compatibility together [102], and human adipose-derived stem cell work cautioned that chitosan–HA matrices alone may not maintain a durable articular chondrogenic phenotype without morphogen support [103].

Recent hydrogel studies moved from simple defect filling toward adhesive, ECM-instructive, drug-conjugated, and granular systems. GelMA–glycol chitosan hydrogels achieved a modulus around 283 kPa, maintained embedded chondrocyte viability above 70%, and improved cartilage adhesion strength from 38 kPa to 52 kPa over 4 weeks, with further improvement to 60 kPa after intermittent mechanical stimulation [104]. Silk fibroin/decellularized cartilage ECM/chitosan hydrogels and methacrylated chitosan–decellularized ECM hydrogels supported BMSC adhesion, aggregation, chondrogenic differentiation, and in vivo cartilage repair in rabbit and goat models [105,106]. Kartogenin-functionalized chitosan methacrylate hydrogels provided visible-light in situ gelation, sustained drug release, and cartilage defect repair [107]. Chitosan nanoparticle and osteoarthritis-oriented hydrogels extended this logic toward intra-articular disease-modifying delivery, including chitosan nanoparticle strategies for osteoarthritis and pH-responsive oxidized hyaluronic acid/quaternized chitosan hydrogels releasing diacerein for up to 47 days in vitro and more than 31 days in rabbits [108,109]. The newest systems include catechol-functionalized chitosan hydrogels with bioadhesion around 1150 kPa and compressive modulus around 195 kPa, chitosan/GelMA granular hydrogels with 6.6-fold cell-number increase after 28 days, and cartilage-mimetic grape-seed-protein/chitosan hydrogels with compressive strength of 42 MPa and friction coefficient of 0.018 [69,70,71].

#### 4.2.2. Osteochondral, Multiphasic, and Matrix-Reinforced Systems

Scaffold architecture strongly influenced chondrogenic output, with fibrous and multilayered chitosan–gelatin scaffolds producing stronger matrix outcomes than less organized structures, even at similar viability. Ragetly et al. reported MSC viability above 90% across chitosan scaffolds but superior GAG and collagen II mRNA in fibrous constructs; Rajagopal et al. found multilayered chitosan–gelatin scaffolds gave approximately 400-fold *COL2A1* upregulation and 1.39-fold higher GAG/DNA than randomly aligned scaffolds, supporting hyaline-like repair in rabbit osteochondral defects [110,111]. Matrix reinforcement amplified these effects: a citric acid-modified chitosan scaffold with PLGA short fibers and cartilage-decellularized matrix increased compressive strength by 349%, Young’s modulus by 153%, GAG by 42%, and type II collagen by 295% [112]. Chitosan–agarose scaffolds supported Wharton’s jelly MSC chondrogenesis with BMP-2 and TGF-β3, reaching 12.71 ± 1.0 µg GAG/µg DNA, while genipin-crosslinked collagen/chitosan scaffolds showed that chitosan ratio and crosslinking tune morphology, pore size, swelling, biostability, and dynamic compression [113,114].

Osteochondral repair required spatially organized cartilage and bone compartments. Hydroxyapatite/chitosan bilayered scaffolds gave an early template for separate chondral and subchondral support, while gene-activated bilayers used plasmid TGF-β1 in the chitosan–gelatin chondrogenic layer and plasmid BMP-2 in the hydroxyapatite/chitosan–gelatin osteogenic layer to support simultaneous cartilage and subchondral bone regeneration [115,116]. Integrated and iterative-overlaying constructs showed that chitosan can contribute to multiphasic interface modeling when combined with hyaluronic acid, collagen, hydroxyapatite, and nano-HAp [117,118]. However, results were not uniform: a magnesium-doped hydroxyapatite/collagen/chitosan scaffold failed to provide superior osteochondral regeneration and showed integration and residual-scaffold limitations, emphasizing that degradation, integration, and subchondral response must be optimized together [58].

#### 4.2.3. Chitosan–Blood Implant Mechanisms and Large-Animal Limitations

Chitosan–glycerol phosphate/blood implants represent a distinct cartilage-repair mechanism. Rather than serving as permanent scaffolds, these systems stabilize marrow-derived clots and modulate early osteochondral wound healing after drilling or microfracture. In ovine and rabbit models, chitosan–GP/blood implants improved early clot adhesion, inhibited clot retraction, increased repair-tissue integration, and promoted more hyaline-like repair tissue compared with bleeding or microfracture controls [119,120]. Mechanistic rabbit studies showed that chitosan–GP/blood implants increased marrow-derived stromal cell recruitment, transient vascularization, intramembranous bone formation, subchondral bone remodeling, neutrophil recruitment, alternatively activated macrophage activity, osteoclast activity, and repair-tissue integration [121,122,123]. Longer-term rabbit data showed that thrombin-solidified chitosan–blood implants improved structural integrity, GAG staining, and type II collagen staining after 6.5 months, supporting persistence of the osteochondral repair effect beyond the early inflammatory phase [124].

The chitosan–blood implant response depended on molecular weight, degradation, drill-hole geometry, species, and defect size. Sheep Jamshidi biopsy and rabbit microdrill studies separated subchondral bone-channel architecture from implant biology, showing that marrow-stimulation geometry strongly influences chondrogenic repair from the bone compartment [125]. In aged rabbits, 150, 40, and 10 kDa implants at 80% degree of deacetylation all recruited neutrophils, osteoclasts, and marrow-derived stromal cells, but the 150 kDa implant degraded less and elicited more apoptotic neutrophils and bone resorption than the 10 kDa [126]. A separate aged-rabbit study showed a more favorable “wound bloom” with 10 kDa than 40 kDa implants, supporting a narrow formulation window for marrow-stimulation augmentation [127].

Large-animal translation was more challenging and is essential for balanced interpretation. In 10 × 10 mm sheep critical-size chondral defects, a rapidly degrading presolidified 10 kDa chitosan–blood implant did not improve resurfacing versus blood implant controls; repair tissue remained inferior to intact cartilage, with approximately two-fold lower GAG and fibril modulus and approximately 4.5-fold higher permeability [59]. In Göttingen minipigs, BST-CarGel (CARGEL Bioscaffold in that model) plus marrow stimulation increased fibrocartilage proportion, 80% versus 64%, and improved some subchondral micro-CT metrics, but hyaline cartilage appeared in only one treated defect, and broad superiority was not established [128]. A 3D in vitro microfracture model further suggested that marrow-derived cells, rather than chondrocytes alone, contribute substantially to new cartilage matrix after chitosan-scaffold augmentation [129].

#### 4.2.4. Preclinical Cartilage Synthesis

Domain synthesis. The supported signal is that chitosan scaffolds and injectable hydrogels can sustain chondrogenic matrix retention and that chitosan–blood implants can stabilize the marrow-stimulation clot, with representative anchors including 87–90% chondrocyte viability in photocrosslinked MeGC/HA gels and bioadhesion near 1150 kPa in kartogenin-loaded systems. What the preclinical evidence does not yet support is hyaline-quality resurfacing of large load-bearing defects, since a rapidly degrading 10 kDa chitosan–blood implant failed to outperform blood implant controls in a sheep model. The main translational bottleneck is matching molecular weight, degradation rate, and defect and surgical geometry to the target lesion rather than demonstrating generic chondrosupportive behavior.

The preclinical cartilage and osteochondral evidence supports four conclusions. First, chitosan can support chondrocyte/MSC attachment, proteoglycan production, collagen II expression, and chondrogenic differentiation, but early porous scaffolds suffered from limited cell penetration and uneven matrix distribution. Second, injectable, photocrosslinkable, and adhesive hydrogels improved defect conformability, cell encapsulation, delivery, and integration, but require a careful balance of modulus, gelation, adhesion, degradation, and cytocompatibility. Third, outcomes improved when chitosan was combined with biologically relevant partners (hyaluronic acid, gelatin, collagen, chondroitin sulfate, agarose, GelMA, decellularized cartilage matrix, HAp, nano-HAp, or growth factors). Fourth, chitosan–blood implants are a biologically distinct marrow-stimulation adjunct whose success depends on molecular weight, degradation, clot stabilization, drill-hole geometry, subchondral remodeling, species, and defect size.

The strongest preclinical message is therefore not that chitosan universally regenerates cartilage, but that it can improve repair when formulation is matched to the biological and mechanical problem: pore architecture, cell seeding, and signaling for porous scaffolds; injectability, gelation, modulus, viability, adhesion, and controlled release for hydrogels; multiphasic spatial design for osteochondral scaffolds; and marrow-clot stabilization for chitosan–blood implants, where large-animal evidence shows formulation and surgical geometry can determine success or failure.

### 4.3. Clinical Cartilage Repair: BST-CarGel/Chitosan–Blood Implant Translation Across Knee, Talus, Hip, and Patellofemoral Lesions

Clinical cartilage repair, particularly BST-CarGel/chitosan–blood implant augmentation of knee marrow stimulation, represented the strongest available human clinical evidence for orthopedic chitosan. Unlike the predominantly preclinical bone, coating, osteomyelitis, cement, tendon/rotator cuff, and disk domains, cartilage repair includes randomized evidence, 5-year follow-up, human biopsy data, joint-specific cohorts, technical implementation papers, safety reports, and health-economic modeling. BST-CarGel should therefore be interpreted as a structurally validated marrow-stimulation adjunct in the knee rather than proof of generalized symptomatic superiority across all cartilage indications.

The clinical evidence is nevertheless joint-specific. Knee evidence is strongest (multicenter randomized controlled trial, 5-year MRI follow-up, human osteochondral biopsy substudy); hip and patellar/patellofemoral applications are promising but mainly observational; and talus/ankle studies show feasibility and symptomatic improvement without consistent superiority over microfracture alone or hyaluronan-based comparators. The evidence is therefore strongest for structural knee repair, not generalized clinical superiority across all joints.

#### 4.3.1. Knee Cartilage Repair: Structural Clinical Signal and Patient-Reported Outcome Caveat

The strongest human evidence for chitosan in orthopedics comes from the international multicenter randomized controlled trial of BST-CarGel plus microfracture versus microfracture alone in femoral condyle cartilage lesions. Stanish et al. randomized 80 patients aged 18–55 years across 26 clinical sites to BST-CarGel plus microfracture (*n* = 41) or microfracture alone (*n* = 39). At 12 months, blinded quantitative 3D MRI showed superiority for both co-primary structural endpoints: lesion filling was 92.8 ± 2.0% with BST-CarGel versus 85.2 ± 2.1% with microfracture, and mean T2 relaxation time was 70.5 ± 4.5 ms versus 85.0 ± 4.9 ms, respectively. Clinical WOMAC outcomes improved in both groups, but the major treatment signal was structural MRI superiority rather than clear short-term patient-reported outcome superiority [47].

The 5-year follow-up by Shive et al. strengthened this structural interpretation: BST-CarGel maintained significantly better lesion filling and repair-tissue T2 over 5 years, with model-based year-5 lesion filling 93.79 ± 1.16% versus 86.96 ± 2.85% and T2 75.68 ± 5.25 ms versus 90.41 ± 6.56 ms. WOMAC pain, stiffness, and function improved from baseline in both groups, but between-group differences were not consistently significant. The most robust signal is therefore durable structural repair, not proven symptom superiority within the available window [56].

The biopsy substudy by Méthot et al. provided human histological support: second-look arthroscopy and osteochondral biopsy at approximately 13 months in 21 BST-CarGel and 17 microfracture patients gave a mean ICRS macroscopic score of 10.7 ± 2.0 versus 7.6 ± 2.7, complete border-zone integration in 71.4% versus 17.6%, intact smooth macroscopic appearance in 61.9% versus 17.6%, and organized deep-zone collagen in 95% versus 53%. Histology also favored BST-CarGel for surface architecture, cell viability, cell distribution, and collagen organization [57].

Pragmatic comparator evidence is more cautious. Sofu et al. compared chitosan–glycerol phosphate/blood implant with a hyaluronan-based cell-free scaffold in 46 patients with focal knee osteochondral lesions (25 chitosan, 21 hyaluronan; mean lesion 3.3 ± 0.7 cm^2^, follow-up 24.4 months). VAS, Lysholm, Tegner, and MOCART were generally similar between groups, with complete defect filling in approximately 28% of chitosan versus 33.3% of hyaluronan cases. Within the chitosan group, lesions ≤ 3 cm^2^ had better VAS and Lysholm outcomes than larger lesions, suggesting lesion size may influence response [130].

Akmeşe/Özbek compared all-arthroscopic implantation of a chitosan-based liquid scaffold with a hyaluronan-based soft scaffold for symptomatic condylar osteochondral lesions of the knee. This retrospective comparative study evaluated 69 patients with symptomatic condylar osteochondral lesions treated using either a hyaluronan-based scaffold (*n* = 37) or a chitosan-based scaffold (*n* = 32). Both groups showed significant improvement in IKDC, Lysholm, and VAS scores at 3 and 12 months postoperatively, but no further significant improvement was observed at 24 months compared with 12 months. No significant differences were found between the two groups in clinical scores or MOCART results, suggesting that both scaffolds are effective for cartilage regeneration, but neither is superior to the other [131].

Taken together, the knee BST-CarGel/chitosan–blood implant is the benchmark for orthopedic chitosan clinical translation. The evidence supports improved structural repair quality and quantity compared with microfracture alone, with convergent MRI, arthroscopic, and biopsy findings. The main limitation is that patient-reported outcome superiority is less consistent than structural superiority, and current evidence should not be interpreted as proof of long-term prevention of osteoarthritis, return-to-sport superiority, or arthroplasty avoidance.

#### 4.3.2. Talus and Ankle Cartilage Repair: Mixed Comparative Evidence

Talar osteochondral lesions differ from femoral condyle defects in cartilage thickness, loading geometry, subchondral architecture, vascularity, containment, and lesion biology, so talus evidence should not be extrapolated directly from knee BST-CarGel data.

Vilá y Rico et al. described arthroscopic bone marrow stimulation plus chitosan–glycerol phosphate/blood implant for talar osteochondral lesions, most useful as technical implementation evidence: arthroscopic BST-CarGel preparation, mixing with autologous whole blood, and application after microfracture to increase clot quantity and residency [132].

Comparative talus data remain mixed. Akmeşe et al. compared a chitosan-based liquid scaffold with a hyaluronan-based soft scaffold in 81 talus patients (42 hyaluronan, 39 chitosan); both improved, but no clear chitosan superiority was shown for AOFAS, VAS, or MOCART [64]. Camurcu et al. compared microfracture plus chitosan–glycerol phosphate/blood implant with microfracture alone in 63 patients (32 chitosan, 31 microfracture; follow-up 32 ± 13 months); both improved, but global clinical and MRI superiority was not demonstrated, with only VAS function favoring chitosan while MOCART parameters did not reach significance [63].

Dhaliwal et al. added further ankle/talus experience with arthroscopic BST-CarGel, best interpreted as supportive feasibility rather than definitive comparative efficacy [133]. On balance, talus evidence supports feasibility and postoperative improvement, but superiority over microfracture alone or hyaluronan-based comparators remains unproven; adequately powered talus-specific trials are needed before knee conclusions can be generalized to the ankle.

#### 4.3.3. Hip, Patellofemoral, and Patellar Cartilage Repair: Observational Extension Evidence

Hip cartilage repair evidence is clinically relevant because acetabular chondral defects are common in femoroacetabular impingement and hip arthroscopy, but the available evidence is mostly observational. Tey et al. described arthroscopic hip application of BST-CarGel with bone marrow stimulation, establishing procedural feasibility rather than comparative effectiveness [134]. Tahoun et al. first reported prospective preliminary outcomes for acetabular chondral delamination associated with femoroacetabular impingement, with clinical improvement and MRI evidence of defect filling in a small cohort [135]. In a later minimum 2-year follow-up study, Tahoun et al. reported 23 patients, mean follow-up 38.4 ± 7.0 months, mean defect size 3.5 ± 1.0 cm^2^, and significant improvement in NAHS, iHOT33, HOS-ADL, and HOS-SSS from baseline. However, femoroplasty corrected the alpha angle from 70.5 ± 6.3° to 44.3 ± 4.9°, making it difficult to isolate the effect of chitosan scaffold augmentation from concomitant correction of femoroacetabular impingement [65].

Safety and imaging studies add context but remain non-randomized. Rhee et al. reported 37 hips treated with BST-CarGel adjunctive to microfracture, with no major device-related adverse events but 2 patients (5.4%) readmitted early for pain with elevated inflammatory markers and negative cultures, suggesting a possible sterile inflammatory reaction [136]. John et al. compared acetabular microfracture plus BST-CarGel with microfracture alone in 80 patients: the CarGel group had larger defects but a lower conversion signal to total hip arthroplasty and less joint-space narrowing, though adjusted clinical-score superiority was less definitive [137]. Tahoun et al. provided T2 mapping in 21 patients and 189 regions of interest, with repair-tissue T2 close to native cartilage [66].

Patellofemoral and patellar evidence is smaller but relevant because this compartment is mechanically challenging. Calvo et al. studied 15 full-thickness patellofemoral lesions, reporting Kujala improvement of approximately 19 points and a mean MOCART 2.0 of 67.67 at 33.36 months [138]. Poggi et al. evaluated 14 isolated patellar lesions at a minimum 2-year follow-up: IKDC improved from 46.2 ± 19.3 to 74.1 ± 23.2 at 24 months, with complete filling in approximately 70%, and MOCART 2.0 of 71.5 ± 13.6 [67]. De Marziani et al. extended this to mid-term follow-up in 13 patients (final 80.2 ± 14.7 months), with IKDC improving from 46.3 ± 20.0 to 70.1 ± 21.5 and final MOCART 2.0 of 72.4 ± 12.5 [68].

In aggregate, hip and patellofemoral/patellar evidence suggests feasibility, clinical improvement, and imaging support but remains lower-level than knee RCT data: hip outcomes are confounded by femoroacetabular impingement correction, labral treatment, and concomitant procedures, and patellar/patellofemoral cohorts are small and non-randomized. These are joint-specific extension evidence, not proof of generalized clinical superiority.

#### 4.3.4. Surgical Implementation, Safety, Biologic Augmentation, and Health-Economic Context

Clinical translation depends on surgical workflow, safety, biologic compatibility, and cost. Steinwachs et al. described arthroscopic BST-CarGel application with microfracture for knee lesions, showing how the technology could move from open or mini-open toward less invasive arthroscopic delivery [139]—important because a clot-stabilizing product must work within arthroscopic defect preparation, fluid management, containment, and polymer–blood handling time.

Safety evidence was generally acceptable but joint-specific. Knee RCT and follow-up data did not identify a major device-safety signal compared with microfracture, whereas the hip safety series reported two early readmissions for inflammatory pain with negative cultures [136]. These findings suggest that postoperative inflammatory symptoms should be monitored, especially in confined joints or technically demanding arthroscopic environments.

Snow et al. explored combining BST-CarGel with bone marrow aspirate concentrate as a biologic augmentation strategy; this is in vitro feasibility evidence rather than clinical efficacy, testing compatibility of clot stabilization with a progenitor-cell-rich concept rather than patient outcomes [140].

Frappier et al. performed a health-economic evaluation of BST-CarGel adjunctive to microfracture versus microfracture alone in knee repair. Over a 20-year horizon, cumulative incremental cost savings became positive by approximately year 4, with base-case 20-year savings of €6448 per patient and larger savings for larger lesions. Because it depends on assumptions about treatment failure, pain management, and downstream procedures, it is supportive implementation evidence rather than definitive cost-effectiveness proof [141].

#### 4.3.5. Clinical Cartilage Synthesis and Claim Boundaries

Domain synthesis. The supported signal is structural: BST-CarGel/chitosan–blood implant augmentation of knee microfracture improves lesion filling, repair-tissue T2, integration, and collagen organization, supported by randomized, 5-year, and biopsy-level data. What the evidence does not support is generalized clinical superiority, because patient-reported outcomes improved in both arms, and superiority for symptoms, return to sport, osteoarthritis prevention, or arthroplasty avoidance is unproven, while talus, hip, and patellar results remain joint-specific and lower-level. The main translational bottleneck is generating joint-specific, adequately powered comparative trials combining structural and patient-reported endpoints rather than extrapolating knee evidence.

The clinical cartilage evidence supports a clear but narrow conclusion. BST-CarGel/chitosan–blood implant augmentation of knee marrow stimulation has the strongest available human clinical evidence among orthopedic chitosan applications. The consistent and best-supported signal is improved structural repair quality and quantity, supported by quantitative MRI, 5-year structural follow-up, arthroscopic assessment, and human osteochondral biopsy data [47,56,57]. However, patient-reported outcome superiority over microfracture alone is less consistent, and available evidence does not yet prove superiority for return to sport, osteoarthritis prevention, arthroplasty avoidance, or broad long-term symptom outcomes.

Joint-specific interpretation is essential: talus evidence shows feasibility and symptomatic improvement without consistent superiority over microfracture or hyaluronan-based comparators; hip evidence is promising but observational and confounded by impingement correction and labral procedures; and patellar/patellofemoral evidence suggests clinical and MRI improvement but is limited by small, non-randomized cohorts. Clinical translation of chitosan–blood cartilage repair is therefore strongest for structural knee repair and still emerging or mixed in other joints.

Future clinical cartilage studies should combine structural and clinical endpoints. Trials should also be joint-specific rather than extrapolating knee evidence directly to the talus, hip, patella, or patellofemoral compartment.

### 4.4. Bone Regeneration and Mineralized Tissue Repair: Chitosan Composites, Scaffold Architecture, Osteogenic Signaling, and Osteoimmunomodulation

Bone regeneration was the largest non-clinical orthopedic domain in the final evidence base. When bone scaffold, mineralized-tissue regeneration, bone-graft modification, osteogenic delivery, and cell-seeded bone repair studies were considered together, the evidence remained predominantly in vitro and preclinical. Most studies used rat calvarial defects, rabbit radius or femoral defects, tibial models, mandibular or craniofacial defects, dog osseointegration models, ectopic ossification assays, or cell-culture systems rather than load-bearing human orthopedic defects.

The central finding was that chitosan was most effective when used as part of a mineralized, reinforced, biologically functionalized, or disease-specific composite rather than as a standalone scaffold. Chitosan alone often lacked sufficient osteogenic potency or mechanical competence for clinically relevant bone repair, whereas combinations with hydroxyapatite (HAp), nano-hydroxyapatite (nano-HAp), calcium phosphate, bioactive glass, gelatin, collagen, silk fibroin, bacterial nanocellulose, β-glucan, agarose, growth factors, peptides, ions, proteins, small molecules, or stem cells produced more compelling preclinical performance.

#### 4.4.1. Mineralized and Architecture-Controlled Chitosan Composites

The dominant bone-regeneration strategy combined chitosan with HAp, nano-HAp, calcium phosphate, or related mineral phases—biologically intuitive because native bone is an organic–inorganic composite. Chitosan partially mimics the organic phase as a biodegradable, cationic, moldable, cell-compatible matrix, while HAp or calcium phosphate provides osteoconductive mineral structure and stiffness [142].

Early in vivo work established the feasibility of chitosan–HAp composites. Danilchenko et al. implanted coprecipitated chitosan/HAp rods into rat tibial defects, supporting their use as biodegradable osteoconductive scaffolds [143], and Lee et al. provided further in vivo support for chitosan–natural nano-HAp scaffolds [144]. Piszko et al. consolidated this direction in a systematic review that screened 375 records and included 20 in vivo studies, all suggesting potential benefit for bone-defect repair while highlighting heterogeneity in animal models, formulations, and outcomes [72].

Recent mineralized scaffolds moved toward controlled mineral morphology and pore architecture. Zhang et al. developed HAp microtubule/chitosan gradient-pore scaffolds with porosity greater than 80%; the 70% HMT–CS formulation resisted compressive deformation and promoted osteogenesis, potentially via oxidative phosphorylation [145]. Liang et al. developed ultralong HAp microtube/chitosan scaffolds with 100–160 µm pores in 8 mm rat critical-size calvarial defects; at 60 days, BV/TV reached 14.07 ± 0.84% versus 9.74 ± 1.36% for chitosan alone, an approximately 44% relative improvement [48]. Phatchayawat et al. fabricated bacterial nanocellulose–chitosan–gelatin–HAp scaffolds with surface pores of 384.5–457.4 µm, interior pores of 467.5–498.7 µm, and porosity of 66.0–81.4%; HAp improved compressive strength, bioactivity, antibacterial behavior, ALP activity, and matrix mineralization [45].

#### 4.4.2. Organic–Inorganic Composites, Biologic Augmentation, and Scaffold Processing

Many studies used chitosan as one component of broader organic–inorganic scaffolds. Electrospun PHBV/chitosan/HAp nanofibers improved osteoblast proliferation and mineral deposition over PHBV alone, while chitosan/bovine-xenograft scaffolds increased porosity-adjusted mechanical performance relative to chitosan alone [146,147]. Gelatin–chitosan–ceramic scaffolds showed that strength and bioactivity do not always rise together: Dasgupta et al. found the HAp formulation had the highest compressive strength, whereas the bioactive-glass formulation gave stronger MSC proliferation, RUNX2/osteocalcin expression, and new bone formation in a rabbit tibial model [148]. He et al. combined chitosan coating, PTMC/PLLA, oleic-acid-modified HAp, and vancomycin microspheres, showing how chitosan contributes to osteoblast adhesion within a polymer–ceramic–drug architecture [149].

Additional scaffold designs broadened the spectrum: chitosan–alginate hybrid scaffolds as an early cationic–anionic polyelectrolyte design, nacre-mimetic HAp/chitosan/gelatin layered scaffolds, and chiral-modified HAp/chitosan scaffolds, suggesting chirality as another structure–property variable [150,151,152]. Zhou et al. combined silk fibroin/chitosan/nano-HAp with autologous concentrated growth factors and BMSCs; the 4% SF/CS/nano-HAp formulation supported BMSC adhesion, proliferation, osteogenic differentiation, and rabbit radius critical-defect repair, illustrating chitosan’s role as a platform for autologous biologic augmentation [153].

Processing variables also strongly influenced performance. Gonçalves et al. varied chitosan concentration, freezing temperature, genipin crosslinking, and magnetite loading, tuning porosity and strength toward different trabecular-bone-like windows [73]. Fathy et al. compared freeze-dried chitosan foam with physically crosslinked chitosan membrane in rat femoral critical-size defects, finding the foam’s larger mean pore size, 65.42 µm versus 6.44 µm, associated with higher in vivo bone-regeneration scores [154]. Hefzollesan et al. used extrusion-based 3D printing of chitosan/magnesium-doped nano-HAp scaffolds functionalized with icariin, lithium chloride, and naringin [155], and Kazemi et al. added thymoquinone-releasing collagen/chitosan/nano-HAp scaffolds [156]. Together, these show that scaffold performance depends on architecture, interconnectivity, crosslinking, mineral phase, processing, and payload rather than chitosan identity alone.

Cell source studies were heterogeneous. Maglione et al. used adipose-derived stem cells in a chitosan–glycerol phosphate scaffold gel maintained by a resorbable membrane; histology suggested early bone formation, but micro-CT differences were not significant and membrane-associated inflammation was noted [157]. Sukpaita et al. tested a chitosan/dicarboxylic acid scaffold seeded with human periodontal ligament cells in mouse calvarial defects, adding hPDLCs to the broader list of mineralized-tissue cell sources investigated with chitosan matrices [158]. These studies support chitosan’s cell-carrier capacity but also show that cell seeding alone does not guarantee robust bone regeneration.

Additional studies reinforced the importance of processing, surface functionalization, and cell source. Costa-Pinto et al. showed that melt-based chitosan-containing scaffolds support hBMSC colonization, metabolic activity, mineralized matrix deposition, and osteogenic marker expression [159]. Brun et al. evaluated chitosan/HAp hybrid scaffolds and later biofunctionalized chitosan surfaces, supporting mineral reinforcement and surface chemistry in osteoblast interaction [160,161]. Przekora et al. studied a ternary chitosan/β-1,3-glucan/HAp scaffold, while Liu et al. loaded urine-derived stem cells onto chitosan-optimized biphasic calcium phosphate scaffolds for large segmental rabbit defects [162,163]. Da Cunha et al. showed that collagen/chitosan scaffolds support in vivo bone repair, although stem-cell addition did not uniformly improve outcomes [164].

#### 4.4.3. Functionalized, Signaling-Active, and Osteoimmunomodulatory Systems

Modern studies moved from descriptive feasibility toward biointeractive, signaling-active design. RGD modification increased BMSC adhesion to chitosan/HAp scaffolds, reaching 80.7% after 4 h, while nanofibrous HAp/chitosan scaffolds promoted osteogenic differentiation through integrin–BMP/Smad signaling, upregulating Smad1, BMP-2/4, RUNX2, ALP, collagen I, integrin subunits, and nuclear pSmad1/5/8 [165,166]. Carboxymethyl chitosan–sodium alginate networks in magnesium phosphate cement activated FAK-dependent Wnt signaling, and guanosine 5′-diphosphate-crosslinked chitosan/HAp constructs released phosphate cues that increased proliferation, ALP activity, osterix expression, calcium phosphate deposition, and callus formation in a mouse tibial fracture model [167,168]. Cai et al. used mesoporous bioactive glass scaffolds with chitosan microspheres for binary IL-8/BMP-2 delivery [169], and Saber et al. introduced biguanidylated chitosan/PVA electrospun nanofibers improving tensile, compressive, osteogenic, antibacterial, and in vivo calvarial performance [170].

Ion substitution, trace-element delivery, and gene/microRNA strategies added further functional layers. Ressler et al. prepared highly porous calcium phosphate/chitosan composite scaffolds substituted with Sr^2+^, Mg^2+^, Zn^2+^, and SeO_3_^2−^, producing interconnected pores, homogeneous calcium phosphate distribution, degradation-medium stability, and higher osteogenesis-related marker expression in human MSCs [171]. Balagangadharan et al. incorporated nano-zirconium dioxide and miR-590-5p into chitosan/nano-HAp scaffolds, moving chitosan bone scaffolds toward microRNA-assisted osteogenic engineering [172]. Ramesh et al. used chitosan infiltration to improve the mechanical and biological behavior of bovine cancellous bone-derived HAp scaffolds, while Koski et al. explored chitosan-loaded HAp disks for post-tumor resection reconstruction, showing cytocompatibility with fetal osteoblasts and a strong reduction in osteosarcoma MG-63 viability in vitro [173,174].

Osteoimmunomodulation emerged as a particularly important direction. Santos et al. used fibrinogen-adsorbed chitosan scaffolds (0.54 ± 0.10 mg fibrinogen/g after adsorption, 0.34 ± 0.04 mg/g after washing); in rat critical-size defects, these improved bone regeneration and angiogenesis and altered immune-cell profiles [175]. Kazimierczak et al. found chitosan/agarose/nano-HAp scaffolds promoted anti-inflammatory cytokines (IL-4, IL-10, IL-13, TGF-β), supporting M2 macrophage polarization and osteogenic differentiation [176]. Tang et al. used directional freeze-cast double-crosslinked carboxymethyl chitosan/alginate/HAp scaffolds; HAp content of 15–45 wt% influenced pore size, porosity, swelling, Ca^2+^ release, stiffness, and modulus, and 45 wt% improved tibial defect healing at 8 weeks through macrophage polarization, osteogenesis, and angiogenesis [74]. Gallinari et al. added an inflammatory-microenvironment model with simvastatin-loaded chitosan–calcium scaffolds, supporting testing in hostile inflammatory conditions, not only ideal defects [177].

#### 4.4.4. Disease-Specific, Craniofacial, and Guided Bone-Regeneration Evidence

Recent studies targeted disease-specific bone microenvironments, especially osteoporosis and metabolic stress. Mureşan et al. evaluated vitamin D3-loaded chitosan nanostructures in vitro and in ovariectomized rats; the 6.25 ng/mL formulation reached 117.77% cell viability at 72 h, increased calcium nodule deposition, and improved mature bone formation and vascularization [178]. Jiang et al. developed an icaritin-loaded biomimetic mineralized carboxymethyl chitosan/alendronate hydrogel with sustained release for at least 14 days and improved osteoporotic defect repair [179]. Lei et al. used a hydroxybutyl chitosan thermosensitive hydrogel/microgel releasing alendronate for six weeks and BMP-2 for more than 35 days, combining osteoclast inhibition with osteogenic stimulation [77]. Lin et al. targeted iron-overload osteoporotic defects with a genipin-crosslinked chitosan–hyaluronic acid hydrogel loaded with chromium picolinate, which reduced ROS, preserved mitochondrial membrane potential, blocked ferroptosis, restored BMSC migration and osteogenic differentiation, and improved BV/TV in rabbit osteoporotic defects [180].

Selected dental, craniofacial, and guided bone-regeneration studies were retained because they provided relevant mineralized-tissue evidence. Gani et al. combined chitosan gel with crab-shell-derived HAp powder in periodontal regeneration and assessed IL-1 and BMP-2, supporting the broader mineralized-tissue principle that chitosan–HAp materials can modulate inflammatory and osteogenic markers [181]. Gerova-Vatsova et al. systematically reviewed chitosan-modified bone-regeneration materials, including 17 in vitro and six in vivo studies, and found advantages in bioactivity, osteogenic potential, biomineralization, biodegradability, antibacterial behavior, biocompatibility, and bone formation [182]. Tripathi et al. reinforced chitosan–HAp membranes with functionalized multiwalled carbon nanotubes, increasing tensile strength from 5.25 MPa to 17.84 MPa and Young’s modulus from 66.89 MPa to 747.02 MPa while maintaining biological compatibility above 50% cell viability [183]. Xiaojie et al. developed an injectable photothermal dual-crosslinked membrane using methacrylated carboxymethyl chitosan, silk fibroin, and bioactive glass, combining barrier function, osteogenic/angiogenic stimulation, antibacterial activity, and rat skull-defect performance comparable to Bio-Gide^®^ collagen membrane [184]. These studies should not be interpreted as direct load-bearing orthopedic evidence, but they strengthen the mineralized-tissue and guided-regeneration rationale for chitosan-containing constructs.

#### 4.4.5. Bone-Regeneration Synthesis

Domain synthesis. The supported signal is that chitosan performs best as part of mineralized, reinforced, or osteoimmunomodulatory composites, with a representative anchor of BV/TV 14.07 ± 0.84% versus 9.74 ± 1.36% for an ultralong-hydroxyapatite-microtube/chitosan scaffold. What the evidence does not support is chitosan as a standalone bone scaffold, since chitosan alone produced little new bone in critical-size radial defects, whereas gelatin and chitosan–gelatin performed better. The main translational bottleneck is large-animal, load-bearing validation against clinically used bone substitutes rather than small-animal, non-load-bearing, surrogate-endpoint models.

The bone-regeneration evidence supports five conclusions. First, chitosan alone is usually insufficient for robust orthopedic bone repair, becoming more effective when mineralized, reinforced, functionalized, or combined with osteogenic cells or payloads; consistent with this, chitosan alone did not promote considerable new bone in a critical-size radial defect model, whereas gelatin and gelatin–chitosan performed better [60].

Second, architecture and mineral phase are decisive: pore size, porosity, interconnectivity, mineral morphology, stiffness, and degradation govern infiltration, vascularization, osteogenesis, and mechanical competence. Informative quantitative anchors include bacterial nanocellulose–chitosan–gelatin–HAp scaffolds (384.5–498.7 µm pores, 66.0–81.4% porosity), ultralong HAp microtube/chitosan scaffolds (100–160 µm pores, ~44% relative BV/TV improvement), and gradient-pore HMT–CS scaffolds (>80% porosity) [45,48,145].

Third, functionalization increasingly defines the field: RGD modification, sulfated chitosan growth-factor binding, fibrinogen adsorption, ion substitution, microspheres, carboxymethyl chitosan networks, and biguanidylated nanofibers show that modern chitosan bone scaffolds are biointeractive rather than inert fillers [165,169,170,171,175].

Fourth, osteogenesis is increasingly linked to mechanistic pathways and immune regulation—integrin–BMP/Smad, FAK–Wnt, oxidative phosphorylation, phosphate release, M2 macrophage polarization, ferroptosis inhibition, ROS-responsive release, and angiogenic coupling—though most such pathway-level mechanisms were reported in single preclinical studies and should be regarded as preliminary until independently replicated. These represent a more mature experimental phase than early studies relying mainly on viability, ALP staining, or descriptive histology.

Fifth, despite broad and mechanistically sophisticated preclinical evidence, clinical orthopedic bone-defect translation remains limited. Most studies used small or medium animal models, non-load-bearing or craniofacial defects, short follow-up, or surrogate cellular endpoints. Few studies addressed load-bearing segmental defects under clinically realistic mechanical environments. Future bone-regeneration studies should therefore prioritize chemistry-defined chitosan, standardized reporting of molecular weight and degree of deacetylation, and large-animal load-bearing models.

### 4.5. Cross-Cutting Delivery Systems: Growth Factors, Platelet Products, Small Molecules, Antibiotics, Genes, and Exosomes

One of the strongest cross-cutting themes was the use of chitosan as a bioactive delivery platform, not only a scaffold or hydrogel matrix. Its cationic charge, biodegradability, polyelectrolyte-complex formation, gel-forming ability, and compatibility with microsphere or nanoparticle fabrication allow delivery of proteins, peptides, growth factors, platelet-derived biologics, antibiotics, hormones, small molecules, ions, microRNAs, genes, and exosomes. In many studies, chitosan’s main value was therefore controlling dose, local retention, spatial localization, release duration, and biological sequence rather than mechanical support alone.

#### 4.5.1. Growth-Factor and Osteogenesis–Angiogenesis Delivery

Growth-factor delivery was most relevant to bone regeneration and osteochondral repair. Early cartilage work used TGF-β1-loaded chitosan microspheres within porous chitosan scaffolds, with sustained release over approximately 5 days and increased chondrocyte proliferation and matrix production [94]. Bone-focused systems used chitosan microspheres or modified chitosan to deliver BMP-2, VEGF, IL-8, or pro-angiogenic peptides; BMP-2-loaded microspheres gave early proof of concept that chitosan can localize osteoinductive signaling within scaffolds [185,186].

More recent studies moved toward sequence-controlled osteogenesis–angiogenesis coupling. A 2-N,6-O-sulfated chitosan-assisted sequential BMP-2/VEGF system showed that release order matters: BMP-2-first/VEGF-later delivery produced more extensive vascular networks and faster bone transformation than less physiologic patterns [78]. Mesoporous bioactive glass scaffolds with chitosan microspheres for binary IL-8/BMP-2 delivery similarly linked microsphere design to combined osteogenic–angiogenic logic [169], and P28/VEGF systems extended this toward peptide-assisted vascularized bone regeneration [187]. Collectively, chitosan delivery systems should be judged not by sustained release alone but by whether timing matches recruitment, angiogenesis, osteogenesis, and remodeling.

Additional studies broadened the carrier evidence. Lin et al. used hierarchical mesoporous bioactive glass scaffolds with IL-8-loaded chitosan microspheres and BMP-2 delivery to initiate guided bone regeneration through spatiotemporal chemotactic and osteogenic signaling [188]. Wang et al. used BMP-2-encapsulated polysaccharide nanoparticles to modify biphasic calcium phosphate scaffolds, while Yadav et al. evaluated orsellinic-acid-loaded chitosan nanoparticles within gelatin/nano-HAp scaffolds for in vitro bone formation [189,190], reinforcing the need to match carrier design, payload timing, and scaffold context.

#### 4.5.2. Platelet-Derived, Hormone, Ion, and Small-Molecule Delivery

Platelet-derived delivery illustrates chitosan’s ability to retain complex biologic mixtures rather than single recombinant growth factors. Santo et al. developed chitosan–chondroitin sulfate nanoparticles for platelet lysate delivery; the system was non-cytotoxic, achieved high encapsulation efficiency for physiological growth-factor levels, released proteins for more than 1 month, and enhanced osteogenic differentiation and mineralization of human adipose-derived stem cells [75], supporting chitosan’s role as a biologic-retention platform for platelet-derived cues.

Hormone, ion, and small-molecule delivery broadened the carrier function. Irmak et al. incorporated 17β-estradiol-loaded PLGA nanoparticles into chitosan–HAp scaffolds, with nanoparticle diameter around 240 nm, encapsulation efficiency 54%, and prolonged estradiol release depending on loading [191]. Boron-containing chitosan systems supported osteogenic differentiation via trace-element delivery [192], and sinapic-acid-loaded and other phytochemical/antioxidant platforms extended chitosan toward small-molecule regulation of osteogenesis and inflammation [193].

Bisphosphonate delivery was especially relevant to osteoporosis-oriented repair. Moradikhah et al. fabricated alendronate-loaded chitosan nanoparticles by microfluidics, with sizes of approximately 102–215 nm, loading efficiency up to 32.42 ± 2.02%, and pH-sensitive release faster at pH 5.8 than pH 7.4 [76]. Lei et al. developed a hydroxybutyl chitosan thermogel/microgel releasing alendronate for six weeks and BMP-2 for more than 35 days, combining osteoclast inhibition with osteogenic stimulation in an osteoporotic context [77]. These show that chitosan carriers can be designed for disease-specific microenvironments rather than generic bone filling.

#### 4.5.3. Antibiotic, Immunomodulatory, Gene, and Exosome Delivery

Antibiotic delivery overlapped with infection and osteomyelitis applications but remains a cross-cutting carrier function. Vancomycin-loaded chitosan thermosensitive hydrogel/nanoparticle systems achieved encapsulation efficiency 60.1 ± 2.1%, drug loading 24.1 ± 0.84%, and sustained release over 26 days in an osteomyelitis-oriented model [79]. Polymicrobial chitosan nanoparticle systems showed local delivery can target both bacterial burden and biofilm recurrence, though in vivo infection control, cytocompatibility, osseointegration, and hardware mechanics must be evaluated together [80].

Gene, microRNA, and immunomodulatory delivery systems expanded chitosan beyond conventional drug delivery. MicroRNA-loaded chitosan/nano-HAp scaffolds moved the field toward gene-regulated osteogenesis, while inflammation-responsive core–shell microhydrogels combined chitosan-based shells with sustained SDF-1 release and MMP-responsive IL-4 delivery for rotator cuff tendon-to-bone healing [172,194]. In intervertebral disk repair, quaternized chitosan/oxidized starch hydrogels loaded with MSC-derived exosomes showed storage modulus around 200 Pa, gel–sol transition strain around 257.3%, pore size 100–200 µm, exosome size 50–200 nm, and sustained exosome release over 14 days, whereas free exosomes released more than 80% within 1 day [88]. These examples show that chitosan delivery is increasingly being used to regulate the inflammatory phase, progenitor-cell recruitment, matrix production, and microenvironmental stress.

#### 4.5.4. Delivery-System Synthesis

Domain synthesis. The supported signal is that chitosan is a versatile cross-cutting carrier for growth factors, platelet products, ions, small molecules, genes, and exosomes, with sustained release demonstrated across systems, including platelet-lysate nanoparticles releasing protein for over one month and sequential BMP-2/VEGF systems showing release-order-dependent bone formation. What the evidence does not support is that longer release equals better outcomes, because release must match the biological window of the indication. The main translational bottleneck is defining dose, loading efficiency, retained bioactivity, local pharmacokinetics, and therapeutic-window matching with in vivo safety, rather than prolonged release alone.

The cross-cutting delivery evidence supports four conclusions. First, chitosan is a versatile carrier whose cationic charge and polyelectrolyte behavior allow interaction with proteins, peptides, nucleic acids, platelet-derived factors, antibiotics, and small molecules. Second, release duration alone is not a sufficient quality marker: antibiotic delivery must remain therapeutically relevant; growth-factor delivery must match recruitment, angiogenesis, osteogenesis, and remodeling; and exosome or platelet-product delivery must preserve bioactivity after release.

Third, chitosan delivery is most convincing when integrated into an indication-specific format: mineralized scaffolds, microgels, or thermogels combining osteogenic and antiresorptive signals for bone; local antibiotic delivery that preserves osseointegration, corrosion resistance, and hardware mechanics for infection; immunomodulatory or chemotactic delivery aligned with tendon-to-bone healing phases for tendon and rotator cuff; and injectable hydrogels that retain payloads while resisting extrusion for intervertebral disk.

Fourth, delivery studies should report dose, encapsulation efficiency, loading efficiency, release kinetics, retained bioactivity, and degradation behavior. A longer release profile is not automatically superior unless it matches the biology of the target indication. Therefore, future orthopedic chitosan delivery systems should be designed around temporally programmed, clinically relevant, and mechanically compatible release rather than generic prolonged delivery.

### 4.6. Anti-Infective Orthopedic Interfaces: Chitosan-Based Coatings, Osteomyelitis Hydrogels, 3D-Printed Antibiotic Scaffolds, and Biofilm Control

Implant-associated infection and osteomyelitis formed a major non-cartilage translational domain. Chitosan systems here were not designed only to kill bacteria; the most translationally relevant studies combined antibacterial activity, biofilm control, local antibiotic delivery, osteoblast compatibility, osseointegration, corrosion resistance, coating stability, and mechanical safety. This dual antibacterial–osteogenic requirement is essential because an implant coating that is antibacterial but cytotoxic, mechanically unstable, corrosive, or poorly osteointegrative is unlikely to succeed clinically.

The anti-infective evidence can be organized into four design strategies: drug-eluting chitosan coatings on titanium, nanotube or hybrid inorganic–organic coating systems, contact-killing or quaternized chitosan interfaces, and injectable or printed chitosan-based systems for osteomyelitis and infected bone defects. Recent synthesis-level evidence supports this organization: Tardelli et al. reviewed chitosan coatings on titanium-based implants and emphasized antibacterial activity, osteoblast compatibility, coating design, and the need to evaluate surface stability together with biological behavior [195]. Broader antimicrobial chitosan literature also shows that antibacterial behavior depends on molecular weight, degree of deacetylation, charge density, derivative type, pH, formulation, microbial strain, biofilm state, and release kinetics rather than chitosan identity alone [18].

#### 4.6.1. Implant Coating Design: Local Release, Nanotube Reservoirs, and Contact-Killing Surfaces

Early titanium coating studies established chitosan as a local antimicrobial delivery layer. Norowski et al. coated titanium with chitosan loaded with tetracycline or chlorhexidine; tetracycline released approximately 89% over 7 days and inhibited bacterial growth by 95–99.9% without detectable osteoblast or fibroblast toxicity, whereas chlorhexidine released faster with greater early cytotoxicity [49]—illustrating the central trade-off between antimicrobial potency, release kinetics, and host–cell compatibility. Swanson et al. extended this to chitosan–vancomycin coatings on modified titanium, combining vancomycin release with osseointegration-oriented surface engineering [196]. David and Nallaiyan anchored chitosan/gelatin–Sr-HAp scaffolds to titanium by dopamine chemistry and loaded them with vancomycin; the HV2 scaffold loaded 82.45 ± 3.5 µg vancomycin, showed stronger activity against MRSA and MSSA than comparators, and maintained MSC viability and spreading [197].

Nanostructured titanium systems used titania nanotubes as drug reservoirs and chitosan as a biopolymer cap or functional coating. Kumeria et al. introduced biopolymer-coated drug-releasing titania nanotubes combining release control, osteoblast adhesion, and antibacterial activity, with chitosan as part of a reservoir–polymer cap system rather than a simple coating [198]. Pawłowski et al. developed a nanosilver-decorated titania nanotube system with a nanotube inner diameter of 97 ± 12 nm and length 342 ± 36 nm, silver nanoparticles of 88 ± 8 nm, with electrophoretic chitosan/polymer coatings achieving 99.9% *E. coli* elimination while preserving early osteoblast compatibility [199]. Kodama et al. extended quaternized chitosan coatings to 3D-printed titanium alloy intervertebral cages, while Rahnamaee et al. showed nanofibrous carboxymethyl chitosan coatings on titania nanotubes supported bone-cell growth, reinforcing the dual osteogenic/anti-infective rationale [200,201].

Hybrid inorganic–organic coating designs further showed that chitosan can regulate both release and bioactivity. Palla-Rubio et al. integrated chitosan into silica–chitosan hybrid coating chemistry for titanium implants, supporting a sol–gel approach to antibacterial coating stability [202]. Doymus et al. immobilized ciprofloxacin-loaded chitosan microspheres and nano-HAp on titanium; immobilization reduced total drug release to 89%, reduced burst release from approximately 55% to approximately 35%, maintained coating stability for 30 days in PBS, and improved simulated-body-fluid HAp growth over 3 weeks, suggesting that mineral overlays can tune both elution and osseointegration potential [203]. Ao et al. developed collagen I/hyaluronic acid/quaternized chitosan multilayer titanium coatings that combined contact-killing HACC retained on the surface with collagenase-mediated HACC release; the coating inhibited *S. aureus*, MRSA, and MRSE colonization and reduced infection-related bone destruction in a rat femoral implant model [204].

#### 4.6.2. Silver, Antimicrobial Peptide, and Multi-Agent Coating Systems

Silver-containing coatings offered strong antibacterial activity but also highlighted the risk of overinterpreting in vitro results. Xie et al. developed a PDA/HAp/AgNPs/chitosan hybrid coating in which polydopamine and chitosan slowed silver-ion release and reduced toxicity, achieving anti-biofilm efficiencies of 91.7%, 89.5%, and 92.0% against *S. aureus*, *S. epidermidis*, and *E. coli* while supporting osteogenic behavior [50]. Croes et al. provided an essential counterpoint: chitosan–silver and chitosan–vancomycin coatings on additively manufactured porous titanium reduced planktonic and adherent *S. aureus* by up to 4 orders of magnitude in vitro, but in vivo diverged—chitosan–vancomycin reduced infection rate versus chitosan-only, whereas chitosan–silver showed no comparable benefit and aggravated infection-mediated bone remodeling, including osteoclast formation and inflammation-induced new bone [205]. This is one of the most important cautionary findings in the coating literature.

Antimicrobial peptide–antibiotic coatings addressed resistance and biofilm persistence through multi-agent design. Zarghami et al. used chitosan/bioactive glass/vancomycin/melittin coatings against MRSA and VRSA; coatings containing both vancomycin and melittin eliminated planktonic and adherent MRSA and VRSA more effectively than single-agent coatings, while melittin supported MC3T3 proliferation [206]. In a related tetracycline/melittin coating, tetracycline contributed antibacterial activity and increased ALP release, while melittin improved MC3T3 proliferation and MRSA eradication [207]. These support chitosan as a multi-agent coating platform, but translation still requires in vivo infection, cytocompatibility, osteogenesis, coating stability, and mechanical testing.

#### 4.6.3. Osteomyelitis and Infected Bone Defects: Injectable, Nanoparticle, and 3D-Printed Systems

Osteomyelitis and infected bone defects require materials that fill irregular cavities, deliver high local antimicrobial concentrations, degrade without a second operation, and ideally support new bone. Chitosan hydrogels suit this because they are injectable, moldable, biodegradable, and compatible with nanoparticle or thermosensitive release. Tao et al. developed a vancomycin-loaded chitosan thermosensitive hydrogel/nanoparticle system in which quaternary ammonium and carboxylated chitosan improved encapsulation (efficiency 60.1 ± 2.1%, drug loading 24.1 ± 0.84%, release over 26 days); in a rabbit osteomyelitis model, it delivered 10 mg vancomycin per rabbit and supported both anti-infection activity and accelerated bone repair [79].

More recent hydrogel and nanoparticle systems targeted sustained release, osteogenesis, polymicrobial infection, and infected bone repair. Zhang et al. designed a magnesium oxide nanoparticle-coordinated phosphocreatine-grafted chitosan hydrogel loaded with vancomycin; the CMPMg-VCM(50) formulation retained antibacterial activity after 12 days of PBS immersion, was non-toxic to MC3T3-E1 cells, promoted ALP secretion and mineral nodule formation, and protected infected bone from osteolysis in a rat osteomyelitis model [208]. Wang et al. combined vancomycin-loaded calcium sulfate hemihydrate/nano-HAp/carboxymethyl chitosan injectable hydrogels with BMSC sheets for infected bone-defect repair, linking antibiotic delivery with tissue-engineered bone regeneration [209]. Zhan et al. designed a CS-HAp hydrogel with sequential vancomycin/BMP-2 release and degradation behavior over approximately 31 days, illustrating how infection control and osteogenesis can be combined in a single degradable scaffold [210]. Zegre et al. extended infection modeling beyond single-species bacteria by developing chitosan-based nanoparticles that reduced *S. aureus*/*C. albicans* polymicrobial biofilms by up to approximately 90% while maintaining human osteoblast cytocompatibility [80]. Tucker et al. used an innately antimicrobial chitosan hydrogel with fosfomycin-loaded PLA microparticles and reduced *S. aureus* planktonic and biofilm burden in vitro as well as bacterial burden in bone and soft tissue in a rat chronic implant-associated osteomyelitis model [211]. Simpson et al. optimized gentamicin-loaded chitosan nanoparticles for osteomyelitis-oriented antimicrobial delivery, reporting particle sizes in the 100–400 nm range, PDI below 0.5, gentamicin entrapment of 10–65%, inhibition zones of 13–24 mm, and complete CFU reduction between 3 and 24 h against relevant pathogens [212].

Three-dimensional printing added patient-specific geometry and reproducible pore architecture to local antibiotic delivery. López-González et al. developed a degradable hybrid scaffold of 3D-printed PCL and vancomycin-loaded chitosan hydrogel; release followed first-order kinetics, rapid in the first hour, then slower with a plateau within 24 h, and the scaffold inhibited *S. aureus* and *S. epidermidis* while remaining compatible with human bone marrow-derived MSCs without loss of ALP activity or alizarin red staining [213]. It is promising for preclinical infected-defect scaffolds, though the rapid 24 h release suggests longer sustained-release designs may be needed for chronic osteomyelitis.

Additional studies further support combined antibacterial and osteogenic validation. Fischer et al. used electrophoretically loaded titania nanotubes on titanium alloy implants as an antibacterial surface strategy to enhance osseointegration, a relevant nanotube comparator for chitosan coatings [214]. Jafarbeglou et al. developed a silk fibroin/chitosan thiourea hydrogel scaffold with antibacterial and osteogenic activity for chronic osteomyelitis, while Lin et al. reported a 3D-printed chitosan-based pH-responsive dual-function osteomyelitis scaffold [215,216]. Zarghami et al. developed a chitosan/bioactive glass/vancomycin/melittin coating to prevent resistant biofilm formation, extending the multi-agent strategy beyond antibiotic-only approaches [206].

#### 4.6.4. Anti-Infective Interface Synthesis

Domain synthesis. The supported signal is strong in vitro and engineering-level anti-biofilm performance, with anti-biofilm efficiencies above 89% for PDA/HAp/AgNP/chitosan coatings and multi-week vancomycin elution from chitosan thermogels. What the evidence does not support is that in vitro killing predicts in vivo infection control, since chitosan-silver coatings on porous titanium showed strong in vitro reduction yet aggravated infection-mediated bone remodeling in vivo. The main translational bottleneck is integrated in vivo testing of bacterial burden, biofilm recurrence, osseointegration, corrosion, coating stability, and hardware mechanics together.

The anti-infective chitosan literature supports four conclusions. First, chitosan is valuable as a local antimicrobial delivery and interface-modification platform. It can release tetracycline, chlorhexidine, vancomycin, ciprofloxacin, gentamicin, fosfomycin, and antimicrobial peptides from titanium coatings, titania nanotubes, microspheres, hydrogels, nanoparticles, and 3D-printed scaffolds.

Second, the best anti-infective systems are not purely antibacterial; they combine bacterial killing, biofilm suppression, osteoblast compatibility, osteogenic signaling, osseointegration, corrosion resistance, coating stability, and mechanical safety. Strong examples include PDA/HAp/AgNPs/chitosan coatings with anti-biofilm efficiency above 89%, chitosan-coated screws preserving torsional yield strength and pullout force, vancomycin-loaded injectable hydrogels with 26-day release, and dual antibacterial–osteogenic systems for infected bone defects [50,51,79,208,209].

Third, in vitro antibacterial activity is insufficient for translation: chitosan–silver coatings performed well in vitro but failed in vivo and worsened infection-mediated bone remodeling, underscoring the need to test bacterial burden, biofilm recurrence, immune response, osteoclast activity, osteolysis, and new bone formation together [205]. Fourth, clinically relevant hardware testing is essential; chitosan-coated titanium screws were not meaningfully weakened (torsional yield strength 1.70 ± 0.00 vs. 1.76 ± 0.05 Nm; axial pullout 68.66 ± 14.36 vs. 70.33 ± 9.71 N) while improving biofilm resistance and osseointegration markers [51].

Chitosan-based anti-infective orthopedic interfaces are polymer-engineering-rich but clinically immature. Future studies should standardize microbial strain and inoculum, planktonic and biofilm CFU endpoints, elution kinetics, and cytocompatibility. Systems that combine antibacterial action with osteogenesis and mechanical safety should be prioritized over coatings or hydrogels that rely only on short-term in vitro bacterial inhibition.

### 4.7. Chitosan-Modified Bone Cements and Injectable Cement Systems: Injectability, Setting Control, Mechanical Reinforcement, Bioactivity, Antimicrobial Function, and Safety Trade-Offs

Bone cement and injectable cement systems represented a major quantitative materials-engineering domain. This section is important from a polymer-science perspective because the cement literature provides measurable, clinically relevant endpoints: injectability, liquid/solid ratio, setting time, anti-washout behavior, compressive and flexural strength, fracture toughness, polymerization temperature, residual monomer, porosity, degradation, antibiotic elution, antibacterial activity, biofilm inhibition, osteoblast compatibility, and in vivo bone formation.

Chitosan played different roles depending on the cement class. In calcium phosphate cement (CPC), hydroxyapatite (HAp) cement, brushite cement, and magnesium phosphate cement, chitosan generally acted as a rheological modifier, anti-washout agent, mechanical reinforcement phase, resorbable porogen, injectable carrier, or cell/growth-factor-compatible additive. In PMMA-based acrylic cement, chitosan was mainly used to improve hydrophilicity, surface bioactivity, antibacterial behavior, exotherm profile, or contact-killing activity through quaternized derivatives. The evidence was strongly formulation-dependent: CPC–chitosan systems often improved setting and mechanical behavior, whereas PMMA–chitosan systems were more sensitive to trade-offs in elution, residual monomer, aging, and ISO-relevant mechanics.

#### 4.7.1. Calcium Phosphate and HAp Cement Systems: Handling, Setting, Porosity, and Mechanics

Early injectable CPC studies established chitosan as a clinically relevant cement modifier. Leroux et al. examined chitosan among several adjuvants for injectable calcium phosphate cement, emphasizing that any additive must improve injectability without compromising setting, disintegration resistance, or mechanics [217]. Xu et al. developed fast-setting anti-washout CPC scaffolds using chitosan, sodium phosphate, hydroxypropyl methylcellulose, mannitol porogen, and absorbable fibers: hardening time decreased from 69.5 ± 2.1 min for CPC control to 8.2 ± 1.5 min for CPC–chitosan–mannitol and 6.7 ± 1.6 min with added fiber, while flexural strength rose to 4.6 ± 1.4 MPa in the fiber-reinforced formulation versus 1.2 ± 0.1 MPa for CPC–chitosan–mannitol [218].

Liu et al. developed injectable calcium phosphate/chitosan composites from a liquid phase of chitosan, citric acid, and glucose combined with tricalcium phosphate powder; setting times ranged from 5 to 30 min, and increasing citric acid concentration raised compressive strength and bioactivity, supporting tunable moldable injectable paste [219]. Sun et al. provided one of the clearest CPC datasets: adding 20% chitosan reduced setting time from 87 ± 7 min to 13 ± 1 min and increased flexural strength from 4 ± 1 MPa to 14 ± 2 MPa, while acidic dissolution remained similar to control [46]. These establish the main CPC–chitosan advantage: chitosan can shorten setting time, improve anti-washout behavior, and reinforce cement when concentration and liquid/solid ratio are optimized.

Chitosan also functioned as a degradable porogen and remodeling aid. Meng et al. incorporated resorbable chitosan microspheres into CPC; 10 wt% microspheres yielded compressive strength 14.78 ± 0.67 MPa and promoted greater degradation and new bone formation than α-TCP/CPC controls in rabbit femoral condyle defects over 8, 16, and 24 weeks [220]. This uses chitosan as a sacrificial phase to introduce degradable porosity, addressing the limitation that conventional CPC sets strongly but remodels slowly.

Injectable HAp, brushite, biphasic calcium phosphate, and magnesium phosphate systems extended chitosan’s role beyond conventional CPC. Konishi et al. developed an injectable chelate-setting HAp cement using 2.5 mass% chitosan solution, with a setting time of 36.3 ± 4.7 min and compressive strength of 19.0 ± 2.1 MPa, plus osteoblast compatibility and pig tibial osteoconductivity [221]. Lee et al. incorporated a chitosan–alginate polyelectrolyte complex into brushite CPC and identified CPC1, with a liquid/solid ratio of 0.45, as an appropriate injectable formulation that improved setting behavior and bone formation in rabbit femoral head defects [222]. Yu et al. modified magnesium phosphate cement with a carboxymethyl chitosan/sodium alginate gel network; the 2% carboxymethyl chitosan/sodium alginate (CMCS/SA) formulation improved compressive strength, washout resistance, setting time, injectable time, and heat release, while promoting osteoblast attachment, proliferation, osteogenic differentiation, and FAK-dependent Wnt signaling [167]. Rattanachan et al. further broadened this category by developing injectable chitosan/biphasic calcium phosphate bone cement, supporting chitosan-containing cements as moldable bone-repair substitutes beyond classical CPC formulations [223].

#### 4.7.2. Cell-Compatible, Growth-Factor-Loaded, and Osteoinductive Cement Systems

Several studies moved cement from passive void fillers toward cell-compatible osteoinductive matrices. Weir and Xu encapsulated human bone marrow MSCs in alginate beads incorporated into CPC, CPC–chitosan, and CPC–chitosan–fiber scaffolds; after 21 days, live-cell percentage and density in CPC-based constructs matched alginate without CPC, ALP activity increased 8-fold by day 14, and staining/SEM/XRD confirmed apatitic mineral deposition, with chitosan and fibers reinforcing CPC without impairing hBMSC osteodifferentiation [224]. Chen et al. used human embryonic stem cell-derived MSCs on CPC–chitosan–RGD scaffolds, with RGD covalently bonded to chitosan, improving strength, toughness, viability, osteogenic marker expression, and approximately two-fold greater mineral synthesis than CPC–chitosan without RGD [225].

CPC–chitosan also served as a growth-factor delivery scaffold. Weir et al. showed CPC with 15% chitosan had flexural strength 19.8 ± 1.4 MPa versus 8.0 ± 1.4 MPa for conventional CPC, and fracture toughness rose from 0.18 ± 0.01 MPa·m1/2 to 0.23 ± 0.02 MPa·m1/2; adding rhBMP-2 raised ALP activity to 305 ± 111 versus 161 ± 27 for CPC–chitosan without rhBMP-2 and 143 ± 19 for CPC alone [81]. More recently, Cai et al. linked CPC–chitosan to osteoporotic repair via an injectable rhBMP-2-loaded calcium phosphate cement/chitosan composite hydrogel; the rhBMP-2/CPC@CH system showed injectability, self-healing, enzymatic degradation, osteogenic stimulation of BMSCs, increased bone mineral density, and bone volume fraction in an ovariectomized cranial defect model, and antibacterial activity against *S. aureus* and *E. coli* [226].

These systems show that chitosan can convert CPC from a mineral void filler into a biologically active injectable scaffold. However, growth-factor-loaded cements require careful dose control, release characterization, ectopic-bone safety assessment, degradation–bone-formation matching, and indication-specific mechanical testing before clinical extrapolation.

#### 4.7.3. PMMA–Chitosan Systems: Bioactivity, Antibacterial Function, and Mechanical Safety

PMMA cement is widely used in arthroplasty, vertebroplasty, and kyphoplasty, but conventional PMMA is biologically inert, nondegradable, exothermic during polymerization, and susceptible to bacterial colonization. Chitosan has therefore been explored as a PMMA modifier, though PMMA–chitosan systems were more formulation-sensitive than CPC–chitosan systems.

Kim et al. developed HAp–chitosan–PMMA bioactive bone cements with higher water absorbency, weight loss, and porosity than conventional PMMA, lower exothermic temperatures, improved cell attachment/proliferation, and better animal biocompatibility and osteoconductivity than pure PMMA. However, compressive Young’s modulus and ultimate compressive strength were lower than those of PMMA, illustrating the core trade-off: bioactivity can improve while mechanical properties decrease [227]. Zapata et al. evaluated acrylic cement with 0–20 wt% chitosan; chitosan loading ≥15 wt% improved bioactivity and biocompatibility and lowered maximum exotherm temperature, but increased setting time and residual monomer content while decreasing mechanical properties. The authors suggested that 15 wt% chitosan may be more suitable for low-weight-bearing applications such as bone void filling or balloon kyphoplasty rather than high-load cemented arthroplasty fixation [228]. Tavakoli et al. used chitosan/graphene oxide nanocomposite as a reinforcing additive in PMMA; 25 wt% Cs/GO increased compressive strength by 16.2%, compressive modulus by 69.1%, and bending strength by 24.0%, while increasing setting time and injectability, reducing maximum temperature, and improving MG-63 cell viability, growth, and adhesion [229].

Antibacterial PMMA systems used chitosan nanoparticles or quaternized derivatives. Shi et al. investigated chitosan and quaternary ammonium chitosan nanoparticles in PMMA, with and without gentamicin; QCS nanoparticles at 15 wt% achieved approximately a 10^3^-fold reduction in viable bacteria on cement surfaces, retained effectiveness after 3 weeks of immersion, and showed no detectable cytotoxicity while preserving meaningful cement strength [230]. Tan et al. developed HACC-loaded PMMA; in vitro, 26% degree-of-substitution HACC at 20 wt% inhibited antibiotic-resistant staphylococcal biofilm more effectively than gentamicin-PMMA, with gene-expression changes suggesting interference with biofilm virulence machinery [82]. A related study showed HACC-loaded PMMA had lower polymerization temperature, prolonged setting, higher hydrophilicity, greater apatite formation, and improved human marrow-derived MSC attachment, proliferation, and osteogenic differentiation versus gentamicin-PMMA [231]. In vivo, it improved radiographic, gross pathological, histopathological, and culture outcomes in a rabbit tibial metaphysis infection model inoculated with 10^7^ CFU methicillin-resistant *S. epidermidis* [232].

Negative evidence is especially important for PMMA. Tunney et al. tested whether adding chitosan to gentamicin-loaded Palacos R cement would increase gentamicin release and improve prevention of staphylococcal colonization or biofilm. Instead, chitosan significantly decreased gentamicin release, did not improve colonization or biofilm prevention, and reduced compressive and bending strengths below ISO minimum requirements after 28 days in saline [52]. This should remain prominent because it shows that chitosan addition can be harmful when particle form, distribution, concentration, cement matrix, elution, and aging are not optimized.

#### 4.7.4. Cement-System Synthesis

Domain synthesis. Calcium phosphate cement–chitosan systems provide the most consistent cement-level support, particularly for injectability, setting control, and flexural reinforcement, with representative improvements in setting time from 87 ± 7 to 13 ± 1 min and flexural strength to approximately 14–19.8 MPa. By contrast, PMMA–chitosan systems should not be interpreted as universally beneficial: chitosan incorporation into gentamicin-loaded Palacos R reduced antibiotic elution, failed to prevent biofilm formation, and decreased compressive and bending strength below ISO requirements after aging, while higher loading increased residual monomer and degraded mechanics. The main translational bottleneck is therefore not whether chitosan adds bioactivity, but whether the final cement preserves indication-specific elution, residual-monomer, aging, fatigue, exotherm, and ISO-relevant mechanical safety.

The cement evidence supports four conclusions. First, CPC–chitosan systems generally show favorable material effects when chitosan is used at controlled levels to improve setting, injectability, washout resistance, porosity, degradation, and strength. The clearest quantitative examples are the Xu and Sun systems, where setting time decreased from approximately 69–87 min to 6.7–13 min and mechanical properties improved substantially [46,218].

Second, chitosan can convert injectable mineral cements into tissue-engineering platforms: cell-compatible CPC–chitosan maintained hBMSC viability for 21 days with ALP activity up eight-fold by day 14, RGD-functionalized CPC–chitosan doubled mineral synthesis versus non-RGD controls, and rhBMP-2-loaded CPC–chitosan raised ALP activity while preserving improved flexural strength and fracture toughness [81,224,225].

Third, PMMA–chitosan systems are promising but mechanically and clinically riskier: HACC-loaded PMMA gave coherent antibacterial and osteogenic evidence across in vitro biofilm inhibition, gene expression, surface bioactivity, osteogenic compatibility, and rabbit infection validation, but other PMMA–chitosan formulations reduced antibiotic release, increased residual monomer, or compromised ISO-relevant mechanics [52,82,228,231,232].

Fourth, clinical translation requires indication-specific cement safety testing. For low-load bone void filling, injectable CPC–chitosan systems may be attractive because they combine moldability, setting control, degradability, osteoconductivity, and delivery. For high-load arthroplasty fixation, PMMA–chitosan systems should not be advanced unless they demonstrate acceptable bending strength, compressive strength, fatigue resistance, aging stability, and residual monomer safety. The central translational message is that chitosan can improve cement performance only when the formulation preserves the mechanical and elution profile required for the intended clinical indication.

### 4.8. Tendon, Ligament, Enthesis, and Rotator Cuff Repair: Fiber Guidance, Anti-Adhesion Scaffolds, Tendon–Bone Interface Engineering, Bioadhesives, PRP Systems, and Immunomodulatory Hydrogels

Tendon, ligament, enthesis, and rotator cuff repair formed a smaller but mechanistically rich domain. Unlike bone and cartilage scaffolds, which target matrix deposition and mineralized or cartilaginous tissue, tendon and enthesis applications require restoration of anisotropic collagen alignment, gliding function, anti-adhesion behavior, tendon-to-bone integration, fibrocartilage formation, vascular and immune regulation, and biomechanical strength. Chitosan was used here as aligned electrospun fibers, braided textile scaffolds, asymmetric anti-adhesion scaffolds, tubular wraps, bioactive ligament coatings, thermosensitive hydrogels, PRP-retentive implants, catechol bioadhesives, dynamic hydrogels, and inflammation-responsive microhydrogels.

The key translational context is tendon-to-bone healing and rotator cuff repair failure. Current surgical repair often heals through fibrovascular scar rather than restoration of the native graded enthesis. Therefore, a useful chitosan-based tendon or rotator cuff material must do more than support cell viability. It should improve collagen organization, interface fibrocartilage formation, bone integration, early fixation strength, cyclic loading resistance, vascularization, macrophage phenotype, and long-term repair integrity.

#### 4.8.1. Fiber, Textile, and Anti-Adhesion Scaffold Logic

The most intuitive tendon strategy used chitosan-containing fibers to reproduce the aligned extracellular matrix of the tendon. Zhang et al. fabricated aligned chitosan-based ultrafine fibers by stable jet electrospinning; the fibers had a mean diameter of 891 ± 71 nm and promoted tenogenic differentiation of human iPSC-derived MSC-like cells, improving repair in a rat Achilles model [233]. Nowotny et al. addressed processability by producing pure high-grade chitosan multifilament yarns and braided 3D tendon-like scaffolds; the chitosan had 95% deacetylation, viscosity 500 mPas, and molecular weight 350 kDa, human MSCs expanded over 28 days, and larger filament yarns produced higher ultimate stress, showing that tendon scaffolds depend on textile-engineering variables such as filament number, yarn diameter, braid geometry, and sterilization [234].

Chitosan was also used to reduce adhesion and guide tendon-sheath-like repair. Jiang et al. developed a TGF-β3 controlled-release chitosan scaffold with chitosan microspheres and synovial cells for tissue-engineered synovial sheath formation; TGF-β3 release reached approximately 46% over 7 days, supporting synovial-cell attachment and viability [235]. Chen et al. fabricated an asymmetric chitosan scaffold with a compact anti-adhesion layer and a loose porous layer for tendon stem/progenitor cell ingrowth; in vivo, TSPC-seeded scaffolds improved collagen alignment, spindle-shaped cell organization, COL1/COL3 production, and tenomodulin expression in Achilles defects [236]. Yousefi et al. developed a tubular chitosan scaffold loaded with ZnO nanoparticles for rabbit deep digital flexor tendon repair; by 8 weeks, the scaffold was absorbed, adhesions were reduced, and histology showed improved collagen arrangement with fewer blood vessels in the chitosan/ZnO group [237].

These support a concise design rule: chitosan tendon scaffolds should not be judged by porosity or biocompatibility alone. For tendon gliding and tendon-sheath repair, spatial organization is essential—one surface limiting peritendinous adhesion while the opposite surface supports tendon-cell ingrowth, collagen alignment, and matrix maturation.

#### 4.8.2. Tendon-to-Bone and Ligament-Tunnel Interface Engineering

Anterior cruciate ligament reconstruction and tendon-to-bone repair require graft integration within bone tunnels or at the enthesis. In this setting, chitosan was used mainly as a bioactive coating or local delivery layer rather than as a primary ligament substitute. Han et al. developed a layer-by-layer PCL nanofibrous membrane coated with chitosan/hyaluronic acid multilayers and loaded with SDF-1α and BMP-2; the membrane recruited BMSCs, promoted osteogenic differentiation, and improved tendon–bone integration, scar reduction, and biomechanics in a rabbit ACL reconstruction model [238]. Chen et al. used surface-functionalized electrospun poly(3-hydroxybutyrate) membranes and sleeves for ACL reconstruction, with chitosan participating in a surface-functionalization strategy intended to improve fixation, osseointegration, fibrocartilage formation, and tendon–bone interface maturation [239]. Jiang et al. used silver@hydroxyapatite nanoparticles with a chitosan/silk fibroin/PET artificial ligament system for rabbit knee ACL rehabilitation, combining antibacterial silver, osteoconductive hydroxyapatite, and chitosan/silk fibroin surface modification to support osseointegration around an artificial ligament [240].

Injectable hydrogels extended chitosan’s role from coating to local biological modulation. Huang et al. developed a thermosensitive chitosan–gelatin–β-glycerol phosphate hydrogel as a collagenase carrier for tendon–bone healing; in a rabbit tibial tunnel model with 36 rabbits, load-to-failure increased from 14.3 ± 3.9 N in controls to 23.8 ± 8.13 N at 8 weeks, an approximately 66% increase in pull-out strength [53]. Wen et al. encapsulated tendon-derived stem cells in an injectable chitosan hydrogel for rabbit rotator cuff healing; the TDSC/chitosan group showed higher tendon-, cartilage-, and bone-related gene expression, clearer cartilage, denser ordered interface collagen, and higher ultimate load than repair-only controls, though not significantly different from chitosan-only hydrogel [85]. Zhang et al. studied tendon stem cells on a dynamic chondroitin sulfate/chitosan hydrogel, adding a cell–hydrogel strategy [86].

Together, these suggest that chitosan-containing interfaces may support tendon-to-bone integration through chemotaxis, osteogenic stimulation, collagen remodeling, cell delivery, and local hydrogel retention. However, the strongest biomechanical signal remains preclinical and model-specific, and most systems have not been tested under clinically realistic fixation, cyclic loading, tendon degeneration, fatty infiltration, or large-tear conditions.

#### 4.8.3. PRP-Retentive, Bioadhesive, and Immunomodulatory Rotator Cuff Systems

Rotator cuff studies increasingly use chitosan to retain platelet products, improve wet adhesion, and program the inflammatory and vascular microenvironment. Deprés-Tremblay et al. tested freeze-dried chitosan solubilized in autologous PRP in rabbit transosseous rotator cuff repair; chitosan–PRP implants remained resident in tunnels at 1 day, adhered to tendon surfaces, recruited polymorphonuclear cells from 1 to 14 days, improved supraspinatus attachment through greater tuberosity remodeling, and reduced heterotopic ossification without detectable deleterious effects [84]. Chevrier et al. also evaluated chitosan–PRP implants in large-animal rotator cuff healing, supporting that chitosan can increase residence and biological activity of platelet-derived signals at the interface [241]. Zhang et al. developed a PRP–carboxymethyl chitosan–tannic acid composite hydrogel; at 8 weeks in a rat rotator cuff model, PRP-CMCS-TA improved BMD, BV/TV, trabecular separation, maximum load, organization, mineralization, and fibrocartilage formation versus controls [87].

Bioadhesion represents a distinct interface strategy. Fang et al. developed a biocompatible graded 3,4-dihydroxyphenyl chitosan bioadhesive using periodate-modified ion-exchange resin bead filtration to oxidize catechol moieties while removing toxic oxidants before application. The resulting BGC adhesive achieved approximately six-fold higher adhesive strength than commercial tissue adhesives, reaching approximately 1 MPa, which was described as about 20% of current suture repair strength. It also maintained cell viability and increased collagen I and Scx expression [83]. This positions catechol-modified chitosan as a wet-interface material rather than only a scaffold or delivery carrier, although in vivo repair testing and comparison with suture/anchor constructs remain necessary.

The newest rotator cuff hydrogels shifted toward immunovascular programming. Chen et al. designed inflammation-responsive core–shell microhydrogels with a MMP-2-responsive GelMA-MMP/IL-4 core and a chitosan/HA/SDF-1 shell; the shell provided sustained SDF-1 release for MSC recruitment, while the core released IL-4 in response to MMP-2 to promote M2 macrophage polarization and improve fibrocartilage formation in rat rotator cuff repair [194]. Wang et al. developed an injectable lactic-acid-incorporated carboxymethyl chitosan/oxidized hyaluronic acid dynamic Schiff-base hydrogel that inhibited NF-κB signaling, promoted M2 macrophage polarization, increased VEGF expression, stimulated endothelial proliferation and migration, and improved tendon–bone regeneration in a rat acute rotator cuff tear model [242].

#### 4.8.4. Tendon/Rotator Cuff Synthesis and Translational Requirements

Domain synthesis. The supported signal is mechanistically advanced tendon-to-bone biology, with representative anchors including load-to-failure increased from 14.3 ± 3.9 N to 23.8 ± 8.13 N for a collagenase-carrier chitosan hydrogel and catechol-chitosan adhesion near 1 MPa. What the evidence does not support is that biological integration translates into mechanical repair superiority, since an electrospun chitosan-coated graft showed cellular and vascular ingrowth without biomechanical superiority at 8 weeks. The main translational bottleneck is clinically realistic retear, cyclic-loading, anchor/suture-compatibility, and aged or osteoporotic-tendon testing.

The tendon, ligament, enthesis, and rotator cuff evidence supports four conclusions. First, chitosan provides topographical guidance as aligned nanofibers or braided textile scaffolds, where fiber diameter, alignment, yarn architecture, braid geometry, and tensile behavior matter more than pore size. Second, it can act as an anti-adhesion, tendon-sheath-mimetic material in asymmetric, tubular, or controlled-release constructs. Third, it can improve tendon–bone healing as a local delivery and interface-modulation platform for collagenase, SDF-1α, BMP-2, tendon-derived stem cells, PRP, or immunomodulatory signals. Fourth, rotator cuff repair is moving toward multifunctional microenvironment engineering—PRP retention, wet adhesion, macrophage polarization, vascularization, fibrocartilage formation, and mechanical-interface reinforcement.

Negative and neutral evidence remains important. In a rat infraspinatus model, an electrospun PCL fiber implant coated with chitosan–PCL graft copolymer showed good cellular infiltration, blood-vessel and tendinous ingrowth, and normal ensheathment histologically; biomechanically; however, force at failure, stiffness, and modulus did not differ from a porous polyurethane implant, repairs typically failed at the surgical site, and median force-at-failure in both implant groups was lower than suture-only repair [243]. Biological ingrowth thus does not necessarily translate into superior mechanical repair strength.

Tendon and rotator cuff chitosan systems are promising but remain predominantly preclinical. The most convincing quantitative signal is the rabbit tendon–bone hydrogel/collagenase study showing an approximately 66% increase in load-to-failure [53]. The most innovative directions are catechol-modified bioadhesives, PRP-retentive hydrogels, tendon-derived stem cell/chitosan hydrogels, and immunomodulatory core–shell microhydrogels. Translation requires clinically realistic fixation constructs, anchor and suture compatibility, aged or degenerative tendon models, large-tear or retear-like models, cyclic loading, standardized failure load and stiffness testing, fibrocartilage quantification, collagen orientation, macrophage phenotype, vascularity, and long-term repair-integrity outcomes.

### 4.9. Intervertebral Disk and Spine Applications: Nucleus Pulposus Supplementation, Annulus Fibrosus Repair, Whole-Disk Constructs, Gene/Cell Therapy, Exosome Delivery, and Translational Biomechanical Models

Intervertebral disk applications formed a distinct and relatively advanced preclinical-translational domain. Compared with cartilage, bone, coating, cement, and tendon applications, the disk literature used different design targets: injectability through clinically realistic needles, in situ gelation, proteoglycan retention, nucleus pulposus-like matrix production, annulus fibrosus repair, disk-height restoration, T2 MRI preservation, extrusion resistance, compressive mechanics, radiopacity, and large-animal delivery feasibility. The field evolved from early cell-compatible chitosan gels toward mechanically reinforced hydrogels, cadaveric and goat-model testing, annulus-specific matrices, exosome-loaded hydrogels, and microenvironment-responsive intradiscal drug delivery.

#### 4.9.1. Nucleus Pulposus Supplementation and Thermosensitive Chitosan Hydrogels

The biological rationale for chitosan in nucleus pulposus repair is its cationic chemistry: nucleus pulposus tissue is rich in negatively charged proteoglycans, especially aggrecan, whose glycosaminoglycan chains maintain hydration and osmotic swelling pressure. Roughley et al. showed that nucleus pulposus cells encapsulated in chitosan-based hydrogels produced newly synthesized anionic proteoglycans retained within the gel rather than released, although annulus fibrosus cells were less consistently supported [244]. Alini et al. provided earlier context for treating disk degeneration as matrix restoration rather than only mechanical stabilization [245]. More recent culture studies by Tang et al. and Yeşilyurt et al. supported chitosan-based matrices for long-term nucleus pulposus-like culture and injectable chitosan/maltodextrin/microcrystalline cellulose hydrogel design [246,247].

Foundational injectable hydrogel studies established feasibility and key formulation variables. Mwale et al. identified 2.5% Protasan UP G213 crosslinked with 5% genipin as a candidate that gelled rapidly at 37 °C, maintained approximately 95% disk-cell viability, produced no inflammatory reaction after subcutaneous implantation in mice, and could be injected into degenerated human cadaveric nucleus pulposus clefts without annular leakage [248]. Richardson et al. showed human MSCs cultured for 4 weeks in chitosan–glycerophosphate hydrogels adopted a nucleus pulposus-like phenotype without osteogenic or hypertrophic collagen X expression [249]. Cheng et al. improved chitosan–β-glycerophosphate hydrogels with gelatin, achieving sol–gel transition at 31.1–33.8 °C, shorter gelation time, and improved gel strength, nucleus pulposus cell viability, proliferation, and sulfated GAG production versus monolayer culture [250].

Later hydrogel studies emphasized ECM mimicry, mechanical tuning, and disease-like culture conditions. Ghorbani et al. developed a multicomponent injectable hydrogel containing chitosan, β-glycerophosphate, hyaluronic acid, chondroitin-6-sulfate, type II collagen, gelatin, and silk fibroin; the formulation was injectable at 4 °C, initiated gelation at 37 °C in approximately 30 min, showed stable viscoelastic behavior, and was cytocompatible with nucleus pulposus cells [251]. Alinejad et al. focused on mimicking human nucleus pulposus mechanics while maintaining injectability and nucleus pulposus cell viability, emphasizing that cytocompatibility alone is insufficient if the hydrogel does not approximate native disk mechanics [252]. Adoungotchodo et al. modified chitosan hydrogels with gelatin and Link N; under degenerative-medium conditions, Link N significantly increased GAG production, reaching a 4.7-fold increase over control and approaching the TGF-β positive control, showing that disease-like biochemical environments should be considered during disk hydrogel testing [253].

#### 4.9.2. Mechanical Restoration, Extrusion Resistance, and Large-Animal Translation

Mechanical reinforcement became central because simple chitosan hydrogels are often too soft for the loaded disk. Doench et al. developed injectable formulations containing 1.7–3.3% *w*/*w* chitosan and 0.02–0.6% *w*/*w* cellulose nanofibers; the 2% chitosan + 0.3% cellulose nanofiber formulation remained injectable under high shear, gelled in situ after intradiskal injection, showed no canal leakage, restored disk height and viscoelastic behavior in pig and rabbit spine models, and achieved dissipative ratio values greater than 0.55 [254]. Huang et al. developed a rapidly in situ-forming chitosan/PEG hydrogel by photocrosslinking and Schiff-base chemistry; the CSMA-PEGDA-L hydrogel had stronger compressive strength than single-crosslinked controls, low nucleus pulposus cytotoxicity, and slowed puncture-induced degeneration in a rat tail model by physically plugging the annular defect [255].

The most advanced translational disk hydrogel line was the dextran–chitosan–teleostean (DCT) triple-interpenetrating-network hydrogel. Smith et al. showed the DCT hydrogel reached approximately 90% of its steady-state aggregate modulus within 10 h, had confined and unconfined compression comparable to native human nucleus pulposus, and did not extrude after 10,000 loading cycles in human cadaveric spine segments after nucleotomy; nucleus pulposus cells remained viable over 14 days, and MSCs were viable with chondrogenic matrix over 42 days [54]. Gullbrand et al. translated DCT into a goat model: ex vivo injection into degenerated lumbar motion segments restored range of motion and neutral-zone modulus toward physiological values, and radiopaque DCT with zirconia nanoparticles was delivered in vivo without impairing nucleus pulposus or MSC viability [55]. These make the DCT line the strongest non-cartilage preclinical-translational chitosan example, connecting injectability, mechanics, extrusion resistance, radiopacity, and large-animal delivery feasibility.

#### 4.9.3. Cell-, Gene-, Exosome-, and Small-Molecule-Responsive Disk Systems

Chitosan hydrogels also served as carriers for cell, gene, growth-factor, exosome, and drug-delivery strategies. Sun et al. tested *Sox9*-transduced autologous BMSCs delivered in thermogelling chitosan–glycerophosphate hydrogel in a rabbit disk degeneration model. BMSCs survived in vivo for at least 12 weeks, and the *Sox9*-BMSC/chitosan group showed better T2-weighted MRI signal, T2 value, nucleus pulposus structure, histology, and immunohistochemistry than controls, positioning chitosan as a gene-enhanced cell-delivery carrier rather than only a hydrogel filler [256]. Ma et al. combined decellularized nucleus pulposus matrix, chitosan hydrogel, nucleus pulposus stem cells, and GDF5-loaded PLGA microspheres. In a rat tail puncture-induced degeneration model, this NPSC-loaded DNPM/chitosan/GDF5 microsphere composite promoted nucleus pulposus regeneration and showed stronger repair effects than BMSC reference strategies [257].

Exosomes and small-molecule systems further shifted the field toward biologically programmed intradiskal therapy. Guan et al. developed an injectable self-healing quaternized chitosan/oxidized starch hydrogel (QCS-OST) loaded with MSC-derived exosomes; it had a storage modulus of around 200 Pa, gel–sol transition strain around 257.3%, pore size 100–200 µm, and encapsulated exosomes of approximately 50–200 nm. Free exosomes released more than 80% within 1 day, whereas QCS-OST/Exos released gradually over 14 days, and in a rat puncture-induced degeneration model, improved disk-height index, T2 MRI signal, proteoglycan/ECM preservation, and senescence/catabolic markers [88]. Chen et al. incorporated lovastatin-loaded nanoparticles into a thermosensitive chitosan/gelatin/glycerophosphate hydrogel for sustained release in disk degeneration [89]. Huang et al. developed injectable chitosan microspheres with manganese dioxide nanozymes and celecoxib-loaded Pluronic micelles to scavenge free radicals, reduce oxidative stress and inflammatory cytokines, and preserve nucleus pulposus hydration and annulus fibrosus lamellar structure in vivo [258]. Hu et al. reported a ROS-responsive self-stabilizing dynamic hydrogel of chitosan–phenylboronic acid, aldehyde–β-cyclodextrin, and hyaluronic acid–dopamine; boronate ester cleavage triggered sinigrin release and dopamine polymerization, while dabigatran release supported inflammation regulation and ECM anabolism [90].

#### 4.9.4. Annulus Fibrosus Repair, Whole-Disk Constructs, and Comparative Limitations

Annulus fibrosus repair requires a different design logic because the annulus is anisotropic, fibrous, layered, and mechanically loaded. Shao and Hunter developed alginate/chitosan hybrid fiber scaffolds; wet-spinning and lyophilization produced aligned fibers of approximately 40–100 µm diameter, the scaffold degraded more slowly than alginate alone, showed no cytotoxicity to 3T3 fibroblasts, and supported canine annulus fibrosus cell growth with collagen I, collagen II, and aggrecan-containing matrix [259]. Liu et al. developed genipin-crosslinked decellularized annulus fibrosus matrix/chitosan hydrogels and showed bFGF enhanced collagen I, collagen II, aggrecan, and GAG secretion by annulus fibrosus-derived stem cells [260]. A later Liu et al. study in a rat tail annulus defect model showed formulation ratio mattered: DAFM/chitosan-1 produced approximately 2-fold higher COL1, COL2, and aggrecan expression than DAFM/chitosan-2, whereas DAFM/chitosan-2 produced higher MMP-9 and IL-6, indicating more chitosan is not necessarily better [261].

Whole-disk reconstruction remained less developed but conceptually important. Yuan et al. created a whole tissue-engineered intervertebral disk using PBST fibers for the annulus fibrosus compartments and chitosan hydrogel for the nucleus pulposus compartment. After staged implantation in nude mice and rabbits, X-ray and MRI showed preservation of intervertebral space height, and cells expressed extracellular matrix, suggesting that chitosan may be best suited to the hydrated nucleus pulposus region while fibrous synthetic polymers may be needed for annular support [262].

Comparative negative evidence is essential. Naqvi and Buckley tested nucleus pulposus cells and bone marrow stem cells in alginate and chitosan hydrogels under disk-like low-glucose, low-oxygen conditions; nucleus pulposus cells remained viable in both, but bone marrow stem cell viability decreased in chitosan, and alginate supported greater sulfated GAG accumulation and collagen II deposition than chitosan. This shows chitosan is not universally superior for disk regeneration and that cell type, hydrogel charge, stiffness, degradation, biochemical environment, and comparator selection strongly influence interpretation [263].

#### 4.9.5. Intervertebral Disk Synthesis and Translational Requirements

Domain synthesis. The supported signal is the most advanced non-cartilage preclinical-translational evidence, including DCT hydrogels reaching approximately 90% of steady-state modulus within 10 h with no extrusion after 10,000 cadaveric loading cycles and goat-model delivery feasibility. What the evidence does not support is human therapeutic efficacy, and chitosan is not universally superior, since alginate supported greater sulfated-GAG and collagen-II accumulation than chitosan for disk cells in one comparison. The main translational bottleneck is long-term large-animal degeneration studies followed by staged first-in-human trials with disk-height-index, T2 MRI, extrusion, annular-integrity, and pain/function endpoints.

The evidence from the intervertebral disk supports five conclusions. First, the cationic charge of chitosan provides a strong rationale for nucleus pulposus supplementation by helping retain newly synthesized anionic proteoglycans within hydrogels. Second, thermosensitive chitosan–glycerophosphate and chitosan–gelatin–glycerophosphate systems support NP-like differentiation and matrix production, but gelation, cytocompatibility, and mechanics must be optimized together. Third, mechanical reinforcement and extrusion resistance are essential; the Doench cellulose nanofiber/chitosan system and the Smith/Gullbrand DCT hydrogel line are the strongest quantitative anchors, with intradiskal gelation without leakage, disk-height restoration, 10,000-cycle cadaveric loading, and goat delivery feasibility. Fourth, modern disk systems are evolving from fillers into biologically programmed carriers for *Sox9*-BMSCs, NPSC/GDF5 microspheres, MSC-derived exosomes, lovastatin nanoparticles, celecoxib/MnO_2_ nanozymes, and ROS-responsive sinigrin/dabigatran delivery. Fifth, annulus fibrosus repair and whole-disk reconstruction require fibrous or decellularized matrix guidance in addition to hydrogel-like nucleus pulposus mechanics.

Despite being one of the most advanced non-cartilage preclinical-translational domains, intervertebral disk chitosan research remains clinically immature. No mature human therapeutic trial evidence is available. Future studies should prioritize long-term large-animal degeneration models, clinically realistic needle delivery, annulus sealing, and hydrogel extrusion resistance.

## 5. Critical Interpretation, Evidence Quality, and Translational Perspectives

### 5.1. Principal Interpretation: Chitosan as a Formulation-Dependent Orthopedic Polymer Platform

The principal interpretation of this review is that chitosan should not be treated as a single orthopedic biomaterial. Across the final evidence base, “chitosan-based” referred to soluble chitosan–blood implants, mineralized porous scaffolds, injectable hydrogels, quaternized antibacterial coatings, nanoparticles, microspheres, aligned fibers, catechol bioadhesives, calcium phosphate cement additives, PMMA cement modifiers, and intervertebral disk hydrogels. These materials differ in molecular weight, degree of deacetylation, derivative chemistry, charge density, architecture, composite phase, crosslinking, degradation, payload, sterilization sensitivity, and mechanical environment.

This formulation-dependent interpretation explains why the strongest clinical evidence is concentrated in knee cartilage repair with BST-CarGel/chitosan–blood implant, whereas most bone, coating, infection, cement, tendon, rotator cuff, and disk applications remain preclinical or translational-preclinical despite promising material and biological performance.

### 5.2. Clinical Translation and Indication-Specific Claim Boundaries

Clinical translation was highly uneven. The strongest available human evidence was for BST-CarGel/chitosan–blood implant augmentation of knee marrow stimulation, supported by randomized evidence, 5-year structural MRI follow-up, and human osteochondral biopsy data; the most consistent signal is improved structural repair quality and quantity versus microfracture alone. However, patient-reported outcome superiority is less consistent, and evidence does not prove long-term superiority in symptoms, return to sport, osteoarthritis prevention, or arthroplasty avoidance.

This distinction between structural improvement and clinical superiority is central. In knee cartilage repair, BST-CarGel improved lesion filling, repair-tissue T2 relaxation, macroscopic repair quality, integration, cell distribution, and collagen organization [47,56,57]. These are meaningful structural and biological signals. However, WOMAC and other patient-reported outcomes improved in both treatment arms, and between-group clinical superiority was not consistently demonstrated. Therefore, the appropriate conclusion is structural clinical benefit in knee marrow-stimulation augmentation, not generalized clinical superiority across all patient-centered outcomes.

Joint-specific interpretation is also essential. Thus, knee evidence should not be extrapolated directly to the talus, hip, patella, or patellofemoral compartment.

Outside cartilage, most applications remain preclinical or translational-preclinical. Tendon/rotator cuff and intervertebral disk applications are mechanistically compelling but remain clinically immature.

The translational maturity framework should therefore be read as an exploratory heuristic, not a formal evidence grade: it distinguishes human clinical evidence from large-animal, cadaveric, small-animal, in vitro, and review-level evidence, but is not a substitute for formal risk-of-bias assessment, guideline grading, regulatory readiness levels, or independent appraisal.

A clinical-readiness summary across orthopedic chitosan applications—including current status, key caveats, and the most important next study for each domain—is provided in Table 5.

### 5.3. Negative and Neutral Evidence as Formulation-Boundary Evidence

Negative and neutral studies are essential because they define the boundaries of chitosan performance. Positive studies show what chitosan can do when formulation, comparator, model, and indication are favorable; negative and neutral studies show when chitosan inclusion is insufficient, poorly matched, mechanically unsafe, or not superior to clinically relevant alternatives.

These studies prevent overgeneralization: chitosan can improve structural cartilage repair in knee marrow stimulation yet fail to resurface larger-animal chondral defects when molecular weight, degradation, defect size, and surgical geometry are poorly matched; support bone formation when mineralized or functionalized yet perform poorly as an isolated scaffold; reduce bacteria in vitro (chitosan–silver) without a favorable in vivo bone response; improve calcium phosphate cement setting and strength yet compromise gentamicin release and ISO-relevant mechanics in some PMMA formulations; and support tendon ingrowth without improving biomechanical repair strength.

Representative negative, neutral, and cautionary findings are summarized in Table 6. The purpose of this table is not to diminish chitosan’s translational potential, but to define formulation boundaries that future studies must address.

Table 6 reinforces the central interpretation of this review: chitosan is not a universally effective orthopedic additive, but a tunable material platform whose success depends on formulation, comparator, mechanics, degradation, release behavior, host response, and indication. Future studies should therefore be designed to define the appropriate chitosan formulation for a specific orthopedic problem instead of demonstrating generic chitosan efficacy.

### 5.4. Evidence Limitations and Reporting Gaps

Several limitations should guide interpretation. First, this was a structured narrative review with descriptive quantitative endpoint extraction, not a systematic review, scoping review, or meta-analysis; PubMed/MEDLINE was the primary database, with Web of Science, Scopus, and Google Scholar as supplementary sources. Source-specific retrieval, duplicate, and exclusion counts were not retained per supplementary platform, which limits reproducibility relative to a formal systematic or scoping review.

Second, screening, domain assignment, maturity scoring, and data extraction were performed by a single investigator; a sequential two-pass methodology improved internal consistency but cannot replace independent cross-validation. To limit interpretation bias, no formal certainty-of-evidence grading was applied; instead, evidence weighting was anchored to reported quantitative endpoints, and both negative and neutral studies were retained to define formulation boundaries. More fundamentally, because this is a single-author narrative review, the studies included, the domains emphasized, and the endpoints extracted necessarily reflect one investigator’s selection and interpretation of a large, heterogeneous literature; the resulting evidence base should therefore be read as a curated, inherently subjective synthesis rather than an exhaustive or independently validated sample, and this selection-and-extraction subjectivity is itself a fundamental limitation of the present review.

Third, reporting of chitosan chemistry was inconsistent: many studies did not fully report source, molecular weight, degree of deacetylation, viscosity, purity, endotoxin status, sterilization, residual crosslinker, derivative chemistry, or batch-to-batch variability—variables that directly influence charge density, solubility, degradation, antimicrobial activity, gelation, cytocompatibility, release kinetics, mechanics, and immune response.

Fourth, models and comparators were heterogeneous across bone (calvarial, tibial, radial, femoral, mandibular, ectopic, and craniofacial), cartilage (rabbit, sheep, minipig, and human, with varied drilling/microfracture geometries), tendon (Achilles, flexor, ACL tunnel, supraspinatus, and rotator cuff), and disk (cells, rat tails, rabbit, human cadaveric, and goat). Empty, untreated, or chitosan-only controls suit proof-of-concept but are insufficient for translation; comparators should increasingly include standard-of-care or best-available alternatives.

Fifth, mechanical qualification was inconsistent outside certain material domains. Bone cement, coated screws, disk hydrogels, and some tendon-to-bone models included useful mechanical testing, but many scaffold and hydrogel studies emphasized viability, marker expression, or histology without sufficient wet mechanical testing, fatigue, degradation-associated strength loss, cyclic loading, extrusion resistance, or failure-mode analysis—important because orthopedic biomaterials face load, motion, fluid flow, shear, and repeated stress.

Sixth, a denominator-based reporting-completeness analysis was not performed because the extraction framework was designed for structured narrative endpoint capture rather than systematic item-level completeness coding across all studies. Therefore, this review identifies recurring reporting gaps qualitatively rather than estimating the proportion of studies reporting each polymer or mechanical variable. Future systematic evidence maps should prospectively code, by domain, the proportion of studies reporting chitosan source, molecular weight, degree of deacetylation, viscosity, purity/endotoxin status, sterilization, residual crosslinker, wet mechanical testing, degradation-associated strength loss, fatigue, release kinetics, cytocompatibility, antibacterial testing, and clinically relevant comparators.

### 5.5. Perspectives and Future Directions: From Chitosan-Containing Materials to Chemistry-Defined Orthopedic Platforms

Future orthopedic chitosan studies should move from proof-of-concept material fabrication toward chemistry-defined, comparator-controlled, mechanically qualified, and indication-specific translational validation. The next phase of the field should be organized around five priorities.

First, chitosan identity must be reported rigorously, following the minimum reporting checklist (Supplementary Table S3) for source, molecular weight, degree of deacetylation, viscosity, purity, endotoxin status, sterilization, derivative chemistry, concentration, crosslinking, and loading; otherwise, two materials both labeled “chitosan-based” may not be scientifically comparable.

Second, biological and mechanical performance should be tested together instead of in isolation, with the specific endpoints for each application—bone scaffolds, cartilage hydrogels, coatings, cements, tendon/rotator cuff systems, and disk hydrogels—provided in the application-specific endpoint checklist (Supplementary Table S5).

Third, future studies should use clinically relevant comparators, not only empty or untreated controls: clinically used bone substitutes, calcium phosphate cements, HAp ceramics, or collagen membranes for bone; microfracture, hyaluronan scaffolds, or collagen membranes for cartilage; antibiotic-loaded materials or standard coatings for infection; and non-chitosan injectable hydrogels such as alginate for the disk.

Fourth, clinical studies should be joint- and indication-specific. Knee BST-CarGel evidence should be extended through long-term registry follow-up, revision-rate analysis, return-to-sport outcomes, osteoarthritis progression, and arthroplasty conversion. Talus studies require adequately powered comparative trials because current evidence does not consistently show superiority over microfracture alone or hyaluronan-based scaffold comparators. Hip and patellar studies require multicenter comparative evidence controlling for concomitant procedures, lesion size, alignment, instability, femoroacetabular impingement correction, and compartment-specific biomechanics.

Fifth, future reporting should be standardized. The minimum reporting checklist (Supplementary Table S3), priority translational studies (Supplementary Table S4), and application-specific endpoint checklist (Supplementary Table S5) are intended to improve reproducibility, cross-study comparison, and translational interpretation. These author-derived tools are proposed to guide future research and to improve the interpretability of a formulation-dependent biomaterial field; they are not externally validated or consensus-developed standards, nor are they meant to impose a systematic-review structure on experimental studies.

Looking beyond chitosan specifically, broader polymer-design work reinforces the case for a chemistry-defined future: recent work on poly(phenylalanine) and poly(3,4-dihydroxy-L-phenylalanine) nanocarriers illustrates how side-chain chemistry, catechol functionality, self-assembly, and stimulus responsiveness can be deliberately engineered into biomedical carriers [265]. The same logic implies that next-generation orthopedic chitosan and chitin-derived systems should be designed, optimized, and reported as chemistry-defined platforms rather than by polymer name alone.

### 5.6. Regulatory, Manufacturing, and Implementation Considerations

Regulatory, manufacturing, and implementation issues should be considered earlier in orthopedic chitosan development. A material that performs well in vitro may fail translationally if the polymer source is inconsistent, sterilization alters molecular weight or viscosity, residual crosslinker remains cytotoxic, degradation products provoke inflammation, batch reproducibility is poor, shelf-life is unstable, or surgical handling is incompatible with operative workflow.

Clinical-grade systems should define acceptance ranges for molecular weight, degree of deacetylation, viscosity, purity, endotoxin, bioburden, residual solvents and crosslinker, heavy metals, protein contamination, and batch variability. Sterilized final products—not only non-sterile prototypes—should be tested for mechanics, degradation, release, cytocompatibility, and biological activity, since sterilization may alter gelation, rheology, coating adhesion, elution, setting time, viscosity, or mechanics.

Combination products require special caution: chitosan systems containing antibiotics, BMP-2, VEGF, SDF-1, IL-4, platelet lysate, PRP, cells, genes, microRNAs, exosomes, silver, or antimicrobial peptides may face different regulatory pathways than scaffold-only materials, since each component adds requirements for dose, release, retained bioactivity, local and systemic exposure, sterility, potency, biodistribution, immune response, resistance risk, and safety.

Surgical workflow is equally important: chitosan–blood implants require autologous blood mixing, defect containment, timing, and stable placement; osteomyelitis hydrogels must fill irregular cavities after debridement, remain in situ, deliver antibiotics, support bone repair, and degrade without removal; tendon and rotator cuff bioadhesives must be compatible with anchors, sutures, arthroscopic delivery, wet surfaces, and early loading; and disk hydrogels must pass through clinically acceptable needles, gel in situ, resist extrusion, and ideally allow radiographic tracking.

The most promising orthopedic chitosan systems will ultimately be those that combine strong biological rationale with controlled polymer chemistry, validated sterilization, scalable manufacturing, reproducible performance, clinically appropriate mechanics, surgical usability, and clear safety profiles. Future development should integrate these requirements at the preclinical design stage, not after efficacy signals have already been generated.

## 6. Conclusions

This structured narrative review, based on descriptive extraction of quantitative endpoints, characterizes chitosan-based biomaterials as a tunable orthopedic polymer platform rather than a single material class. This conclusion is a materials-design and evidence-synthesis framing and should not be read as a generalized claim about the overall clinical potential of chitosan-based biomaterials: as detailed below, direct human clinical evidence is currently confined to a single cartilage-repair technology, while the remaining orthopedic applications remain preclinical or translational-preclinical. Across the final evidence base of 258 unique publications, chitosan performance depended on polymer source, molecular weight, degree of deacetylation, derivative chemistry, cationic charge density, architecture, composite phase, crosslinking, degradation, release kinetics, payload, sterilization, host environment, and target-tissue mechanics.

The strongest available human clinical evidence currently supports BST-CarGel/chitosan–blood implant augmentation of marrow stimulation in knee cartilage repair. Randomized clinical evidence, 5-year structural MRI follow-up, and human osteochondral biopsy data support improved structural repair quality and quantity compared with microfracture alone. However, the best-supported clinical signal is structural repair improvement, not proven long-term superiority in symptoms, return to sport, osteoarthritis prevention, or arthroplasty avoidance. Importantly, this human evidence essentially reduces to a single chitosan–glycerophosphate/blood implant technology and should therefore not be generalized to other chitosan chemistries, derivatives, or formulations, which remain preclinical.

Most non-cartilage applications remain preclinical or translational-preclinical. Bone regeneration has the broadest preclinical evidence, particularly for mineralized, reinforced, functionalized, and osteoimmunomodulatory composites. Implant coatings, osteomyelitis hydrogels, bone cements, tendon/rotator cuff systems, and disk biomaterials show promising material, antibacterial, release-kinetic, biomechanical, or mechanistic evidence but require clinically relevant comparators, mechanical qualification, long-term validation, and indication-specific safety testing before clinical extrapolation.

Negative and neutral findings show chitosan is not a universally effective orthopedic additive; systems may fail when molecular weight, degradation, architecture, payload, elution, mechanics, cytocompatibility, host response, or comparator selection are poorly matched to the indication. Future studies should therefore define which chitosan formulation works, in which defect type, through which mechanism, under which conditions, and against which standard-of-care or best-available alternative.

The main limitation of the orthopedic chitosan literature is the lack of standardization. Progress will depend on rigorous reporting of chitosan chemistry, architecture, mechanics, degradation, release kinetics, immune response, antibacterial performance, comparator selection, sterilization, manufacturing, surgical handling, and translational readiness, moving from “chitosan-containing” materials toward chemistry-defined, mechanically qualified, biologically programmed, comparator-controlled, and indication-specific systems.

## Figures and Tables

**Table 1:** Operational boundary rules for inclusion in the structured narrative evidence base.

Category	Included When	Excluded or Not Interpreted as
Core orthopedic biomaterial studies	Chitosan, chitin, or a chemically modified chitosan derivative was used as a structural, functional, delivery, coating, adhesive, antimicrobial, or cement-modifying component in bone, cartilage, tendon, ligament, enthesis, spine, implant, osteomyelitis, or bone-cement applications.	Studies in which chitosan was only a minor laboratory reagent without functional biomaterial relevance.
Dental, periodontal, and craniofacial studies	The study provided directly relevant mineralized-tissue information, such as bone regeneration, hydroxyapatite/calcium phosphate composites, guided bone-regeneration membranes, osteogenic differentiation, graft bioactivity, or scaffold architecture.	Not interpreted as direct evidence for load-bearing orthopedic defects unless the model or endpoint supported such an interpretation.
Polymer-science and historical sources	The source clarified chitosan identity, source, processing, molecular weight, degree of deacetylation, derivative chemistry, antimicrobial mechanism, characterization, or standardization needed to interpret orthopedic formulations.	Not counted as primary application evidence when assigning translational maturity.
Review-level publications	The review provided background, terminology, polymer-science context, or evidence-landscape support.	Not treated as equivalent to primary experimental or clinical studies for maturity assignment.

**Table 2:** Core chitosan property–performance relationships relevant to orthopedic translation.

Chitosan Variable	Main Material Consequence	Orthopedic Relevance	Reporting/Translation Caveat
Degree of deacetylation/acetylation pattern	Cationic charge density, hydrophilicity, crystallinity, degradation rate, antimicrobial behavior, protein/cell interaction	Antibacterial coatings, injectable hydrogels, chitosan–blood implants, delivery	DDA and acetylation pattern must be reported; otherwise, two “chitosan-based” systems are not scientifically comparable
Molecular weight	Viscosity, injectability, mechanical contribution, degradation rate, release kinetics, immune response	Injectable cartilage/disk hydrogels, particles, fibers, chitosan–blood implants	Low-MW and high-MW chitosan should not be interpreted as one material class (e.g., a 10 kDa blood implant is not a high-MW fiber)
Source, purity, endotoxin/bioburden	Inflammatory and immune response, batch reproducibility, residual protein/heavy-metal load	All implantable and injectable systems	Clinical-grade systems require defined acceptance ranges; endotoxin/bioburden are frequently unreported
Sterilization method	Chain scission, MW/viscosity loss; shifts in gelation, rheology, coating adhesion, setting, and mechanics	All translational systems	The sterilized final product, not the non-sterile prototype, must be re-tested for mechanics, release, and bioactivity
Derivatization chemistry, degree of substitution, substitution pattern	Solubility, contact-killing, growth-factor binding, photocrosslinking, wet-tissue adhesion	CMC, HACC/quaternized, sulfated, methacrylated, and catechol systems	“Chitosan-based” labeling should shift to chemistry-defined reporting; performance depends on substitution degree/pattern and residual reagents
Composite phase and phase ratio	Mechanical strength, osteoconductivity, mineralization, degradation, interfacial compatibility	Bone scaffolds, bone cements, coatings	Chitosan alone is often insufficient for load-bearing bone; phase and ratio must be reported
Crosslinking, residual crosslinker/solvent	Modulus, swelling, degradation, cytocompatibility	Hydrogels, bioadhesives, cements	Residual crosslinker/solvent toxicity and degradation products must be measured, not assumed benign
Final format and architecture	Pore size, porosity, fiber alignment, coating thickness, hydrogel modulus, particle size	Scaffolds, fibers, coatings, hydrogels, particles	Format-specific quantitative endpoints are required and differ by indication

DDA, degree of deacetylation; MW, molecular weight; CMC, carboxymethyl chitosan; HACC, quaternized (hydroxypropyltrimethyl ammonium) chitosan. These variables are interdependent, and Figure 2 presents the same structure–property–performance logic conceptually; two systems labeled “chitosan-based” are scientifically comparable only when these descriptors are reported.

**Table 3:** Domain-level distribution of the final evidence base.

Primary Evidence Domain	Unique Publications, *n*	Proportion of Final Evidence Base, %	Dominant Material Formats/Evidence Types	Main Translational Interpretation
Bone scaffold and mineralized-tissue regeneration	49	19.0	Porous scaffolds, hydroxyapatite/chitosan composites, nano-hydroxyapatite systems, calcium phosphate composites, bioactive glass, gelatin/collagen/silk composites, 3D-printed scaffolds, guided bone-regeneration membranes, osteoporotic bone-defect systems	Largest orthopedic application cluster; broad preclinical evidence but limited mature human orthopedic translation
Cartilage, osteochondral, osteoarthritis and preclinical chondral biomaterials	47	18.2	Porous cartilage scaffolds, chitosan–gelatin scaffolds, chitosan-HA hydrogels, injectable hydrogels, osteochondral bilayer scaffolds, decellularized cartilage matrix systems, kartogenin systems, OA drug-delivery systems	Extensive preclinical and translational-preclinical literature; heterogeneous designs and models
Foundational chitosan polymer science, historical sources and thematic reviews	38	14.7	Chitin/chitosan history, source and processing, molecular weight, degree of deacetylation, derivative chemistry, antimicrobial mechanisms, characterization and standardization reviews	Provides a polymer-science foundation for interpreting orthopedic chitosan as a variable material family rather than a single polymer
Implant coatings, anti-infective interfaces and osteomyelitis systems	26	10.1	Titanium coatings, chitosan antibiotic coatings, silver-containing coatings, titania nanotube systems, antimicrobial peptide coatings, osteomyelitis hydrogels, antibiotic nanoparticles, polymicrobial infection systems	Strong engineering and preclinical infection-control evidence; clinical translation requires in vivo infection, osseointegration and mechanical validation
Intervertebral disk and spine applications	25	9.7	Nucleus pulposus hydrogels, annulus fibrosus scaffolds, thermosensitive hydrogels, DCT hydrogels, exosome hydrogels, drug-loaded chitosan microspheres, annulus sealing membranes	Advanced preclinical-translational evidence, including cadaveric and large-animal work, but no mature human therapeutic trial evidence
Clinical and translational cartilage repair	22	8.5	BST-CarGel/chitosan–blood implants, knee RCT and 5-year follow-up, biopsy studies, hip/talus/patellar cohorts, technique papers, economic analysis	Highest level of human clinical evidence in the final evidence base, especially for knee microfracture augmentation; joint-specific evidence remains heterogeneous
Bone cement and injectable cement systems	21	8.1	Calcium phosphate cement–chitosan systems, PMMA–chitosan systems, quaternized chitosan-loaded PMMA, injectable hydroxyapatite cement, cement–hydrogel composites	Quantitatively rich material evidence; clinical use depends on setting, injectability, ISO-relevant mechanics, elution and aging safety
Tendon, ligament, enthesis and rotator cuff repair	18	7.0	Aligned fibers, braided textiles, anti-adhesion scaffolds, PRP-loaded hydrogels, chitosan bioadhesives, tendon-to-bone hydrogels, rotator cuff anchor coatings	Mechanistically advanced but predominantly preclinical; translation requires clinically realistic fixation and retear models
Cross-cutting drug, growth-factor, nanoparticle and biologic delivery systems	12	4.7	Growth-factor microspheres, platelet lysate nanoparticles, BMP/VEGF delivery, ion delivery, small-molecule delivery, gene/miRNA/exosome systems	Delivery evidence spans multiple orthopedic domains and supports chitosan’s role as a carrier platform rather than only a structural scaffold
Total	258	100.0	-	-

Note: Publications were counted once according to their primary contribution to the review. Some publications contributed to more than one conceptual theme, for example, both scaffold design and delivery, or both infection control and bone regeneration; however, for domain-level distribution, they were assigned to the most specific dominant domain. Detailed study-level extraction is provided in Supplementary Table S1.

**Table 4:** Integrated quantitative performance anchors and translational interpretation across orthopedic chitosan applications.

Application Domain	Highest Available Evidence Level in This Review	Representative Quantitative Anchors	Interpretation
Knee cartilage repair with BST-CarGel/chitosan–blood implant	Highest available human evidence: multicenter RCT, 5-year follow-up, and biopsy substudy	RCT: In total, 80 patients; lesion filling 92.8 ± 2.0% with BST-CarGel vs. 85.2 ± 2.1% with microfracture; T2 relaxation 70.5 ± 4.5 ms vs. 85.0 ± 4.9 ms at 12 months. Five-year follow-up: filling 93.79 ± 1.16% vs. 86.96 ± 2.85%; T2 75.68 ± 5.25 ms vs. 90.41 ± 6.56 ms. Biopsy: ICRS macroscopic score 10.7 ± 2.0 vs. 7.6 ± 2.7; complete integration 71.4% vs. 17.6%; organized deep-zone collagen 95% vs. 53% [47,56,57].	Highest available human evidence in the final evidence base. The best-supported signal is improved structural repair quality and quantity; patient-reported outcome superiority over microfracture alone is less consistent.
Talus cartilage repair	Intermediate human evidence: comparative clinical cohorts and technique papers	Camurcu: In total, 63 patients, 32 chitosan vs. 31 microfracture, mean follow-up 32 ± 13 months; VAS function favored chitosan, but broad clinical and MOCART superiority was not demonstrated. Akmeşe: In total, 81 talus patients, 42 hyaluronan vs. 39 chitosan; no clear superiority for AOFAS, VAS, or MOCART outcomes [63,64].	Feasible and clinically relevant, but superiority over microfracture or hyaluronan-based scaffolds remains unproven. Requires adequately powered talus-specific comparative trials.
Hip and patellofemoral cartilage repair	Emerging human evidence: observational cohorts, safety series, T2 mapping, and small prospective series	Hip: Tahoun reported 23 patients, mean defect size 3.5 ± 1.0 cm^2^, mean follow-up 38.4 ± 7.0 months. Hip T2 mapping: In total, 21 patients and 189 ROIs, repair tissue T2 values close to native cartilage. Patella: IKDC improved from 46.2 ± 19.3 to 74.1 ± 23.2 at 24 months; MOCART 2.0 was 71.5 ± 13.6. Mid-term patellar follow-up reached 80.2 ± 14.7 months [65,66,67,68].	Promising but lower-level evidence than knee RCT data. Hip results are confounded by femoroacetabular impingement correction and labral procedures; patellar cohorts remain small.
Preclinical cartilage, osteochondral, and osteoarthritis biomaterials	Preclinical to translational-preclinical: scaffold, hydrogel, animal model, and OA delivery studies	Injectable methacrylated glycol chitosan (MeGC)/hyaluronic acid (HA) hydrogel: 40 s visible-light irradiation produced stable gels with 87–90% chondrocyte viability; longer irradiation increased modulus but reduced viability. Bioadhesive KGN chitosan hydrogel: bioadhesion ~1150 kPa and compressive modulus ~195 kPa. Granular chitosan/GelMA hydrogels: In total, 6.6-fold cell-number increase after 28 days. Cartilage-mimetic chitosan hydrogels: compressive strength 42 MPa and friction coefficient 0.018 [44,69,70,71].	Rapidly expanding material-design domain. Newer systems emphasize adhesion, modular granular architecture, sustained kartogenin/diacerein delivery, ECM mimicry, and OA microenvironment regulation.
Bone scaffold and mineralized-tissue regeneration	Broad preclinical evidence: in vitro studies, rat/rabbit/dog models, systematic reviews, 3D printing, osteoporotic and inflammatory models	Chitosan/nano-HAp systematic review: 375 records were screened within that review, and 20 in vivo studies were included. HMTs–CS scaffold: pore size 100–160 μm and BV/TV 14.07 ± 0.84% vs. 9.74 ± 1.36% for chitosan alone at 60 days. Gonçalves: porosity > 80% with compressive strength 0.40 MPa and modulus 3.57 MPa in optimized chitosan scaffolds. CAHA immunomodulatory scaffold: 45 wt% HAp improved tibial defect healing at 8 weeks [48,72,73,74].	Largest preclinical orthopedic domain. Evidence supports mineralized, reinforced, osteoimmunomodulatory, and disease-specific chitosan composites, but human orthopedic translation remains limited.
Cross-cutting drug, growth-factor, nanoparticle, and biologic delivery	Preclinical cross-domain evidence: microspheres, nanoparticles, platelet products, small molecules, ions, genes, miRNA, exosomes	Platelet lysate chitosan–chondroitin sulfate nanoparticles released proteins for >1 month. Alendronate-loaded chitosan nanoparticles measured approximately 102–215 nm with loading efficiency up to 32.42 ± 2.02%. Hydroxybutyl chitosan thermogel released alendronate for six weeks and BMP-2 for >35 days. Sequential BMP-2/VEGF systems showed release-order-dependent bone regeneration [75,76,77,78].	Chitosan is strongly supported as a cross-cutting delivery platform across bone, cartilage, infection, tendon/rotator cuff, and intervertebral disk applications. Translation should focus on dose, encapsulation/loading efficiency, release kinetics, retained bioactivity, local pharmacokinetics, therapeutic-window matching, and in vivo safety rather than prolonged release alone.
Implant coatings, anti-infective interfaces, and osteomyelitis systems	Strong preclinical engineering evidence; clinical translation requires in vivo infection, osseointegration, and mechanical validation: in vitro biofilm assays, animal infection models, coated titanium, local antibiotic hydrogels	PDA/HAp/AgNPs/chitosan coating: anti-biofilm efficiencies of 91.7%, 89.5%, and 92.0% against *S. aureus*, *S. epidermidis*, and *E. coli*. Vancomycin-loaded chitosan thermogel/nanoparticles: encapsulation efficiency 60.1 ± 2.1%, drug loading 24.1 ± 0.84%, release for 26 days. Polymicrobial osteomyelitis nanoparticles: up to 90% reduction in *S. aureus*/*C. albicans* biofilms. Chitosan-coated screws preserved torsional yield strength and axial pullout force [50,51,79,80].	Promising for infection control, but in vitro antibacterial results are insufficient. Translation requires simultaneous testing of bacterial burden, biofilm recurrence, osseointegration, inflammation, coating stability, corrosion, and hardware mechanics.
Bone cement and injectable cement systems	Quantitatively rich material evidence: CPC, PMMA, injectable HAp cement, cement–hydrogel composites	CPC–chitosan: setting time reduced from 87 ± 7 min to 13 ± 1 min and flexural strength increased from 4 ± 1 MPa to 14 ± 2 MPa. CPC with 15% chitosan: flexural strength 19.8 ± 1.4 MPa vs. 8.0 ± 1.4 MPa for conventional CPC. HACC-PMMA: 26% degree-of-substitution HACC at 20 wt% inhibited antibiotic-resistant staphylococcal biofilm. Negative PMMA evidence: chitosan decreased gentamicin release and reduced mechanical performance below ISO requirements after aging [46,52,81,82].	Quantitatively rich material domain. CPC–chitosan systems are generally favorable for injectable bone repair and delivery, whereas PMMA–chitosan systems are formulation-sensitive and require ISO-relevant aging, fatigue, elution, residual monomer, exotherm, and long-term mechanical validation.
Tendon, ligament, enthesis, and rotator cuff repair	Mechanistically advanced preclinical evidence: rat/rabbit tendon-to-bone models, fibers, hydrogels, PRP systems, bioadhesives, anchor coatings	Thermosensitive chitosan–gelatin–glycerol phosphate/collagenase hydrogel increased tendon–bone load-to-failure from 14.3 ± 3.9 N to 23.8 ± 8.13 N at 8 weeks. Catechol-modified chitosan bioadhesive reached ~1 MPa adhesive strength, approximately 6-fold above commercial tissue adhesives. Chitosan–PRP implants remained resident in transosseous tunnels and recruited polymorphonuclear cells from 1 to 14 days. Additional tendon/rotator cuff systems used chitosan hydrogels, PRP-retentive constructs, and immunomodulatory interface strategies to support tendon-to-bone healing [53,83,84,85,86,87].	Promising but preclinical. Translation requires clinically realistic retear models, anchor/suture compatibility, aged or osteoporotic tissue models, cyclic loading, and long-term repair-integrity outcomes.
Intervertebral disk and spine applications	Advanced preclinical-translational evidence: in vitro, cadaveric, goat, rabbit/rat models, annulus sealing, and microenvironment-responsive hydrogels	DCT hydrogel reached ~90% of steady-state aggregate modulus within 10 h and showed no extrusion after 10,000 loading cycles in human cadaveric spine segments. Goat DCT work demonstrated ex vivo mechanical restoration and radiopaque in vivo delivery feasibility. QCS-OST/Exos hydrogel released exosomes gradually over 14 days, compared with free exosomes releasing >80% within 1 day. Newer chitosan systems deliver lovastatin, celecoxib/MnO_2_ nanozymes, sinigrin/dabigatran, or annulus-sealing membranes [54,55,88,89,90].	Most advanced non-cartilage preclinical-translational domain. Needs long-term large-animal degeneration studies and eventually human trials with DHI, T2 MRI, extrusion, annular integrity, pain/function, and mechanical loading endpoints.

Note: The table lists selected representative quantitative anchors to support domain-level interpretation. Full study-level extraction data and more detailed quantitative anchors are provided in Supplementary Tables S1 and S2. The highest level of human clinical evidence was found for knee cartilage repair with BST-CarGel/chitosan–blood implant. Most other orthopedic domains remain preclinical or translational-preclinical despite promising material, biological, imaging, release-kinetic, antibacterial, or biomechanical evidence.

**Table 5:** Clinical-readiness summary for orthopedic chitosan applications.

Application	Current Status	Key Caveat	Most Important Next Study
Knee cartilage repair (BST-CarGel)	Clinically supported for structural repair after microfracture	Structural benefit is stronger than proven symptom superiority; no proven prevention of osteoarthritis progression or arthroplasty conversion	Long-term registry or pragmatic cohort assessing revision, return to sport, osteoarthritis progression, and arthroplasty conversion
Talus cartilage repair	Feasible, but not proven superior	No consistent superiority versus microfracture alone or hyaluronan-based scaffold comparators	Adequately powered talus-specific randomized or matched comparative trial
Hip and patellofemoral cartilage repair	Emerging observational evidence	Confounded by femoroacetabular impingement correction, labral procedures, alignment, instability, and small cohorts	Multicenter comparative studies controlling concomitant procedures and lesion characteristics
Bone regeneration	Broadest preclinical domain	Most evidence remains small-animal, craniofacial, non-load-bearing, or surrogate-endpoint-based	Large-animal load-bearing defect studies versus clinically used bone substitutes
Implant coatings and infection-control interfaces	Preclinical engineering evidence	In vitro antibacterial activity does not prove in vivo infection control or osseointegration benefit	Integrated in vivo studies measuring bacterial burden, biofilm recurrence, osseointegration, corrosion, coating stability, and hardware mechanics
Calcium phosphate cement–chitosan systems	Favorable preclinical material evidence	Handling and mechanics are promising but require indication-specific validation	In vivo bone-repair studies with ISO-relevant mechanical, degradation, and fatigue testing
PMMA–chitosan cement systems	Formulation-sensitive preclinical evidence	Elution, residual monomer, aging, fatigue, and ISO mechanical trade-offs are critical	ISO-compliant aging, fatigue, antibiotic-elution, residual-monomer, and infection studies
Tendon and rotator cuff repair	Promising but preclinical	Biological ingrowth does not necessarily translate into mechanical superiority	Clinically realistic fixation, cyclic-loading, retear, anchor/suture compatibility, and degenerative-tendon models
Intervertebral disk biomaterials	Advanced preclinical-translational evidence	No mature human therapeutic evidence	Long-term large-animal degeneration studies followed by carefully staged first-in-human feasibility trials

**Table 6:** Negative and neutral evidence defining formulation-dependent limitations of orthopedic chitosan biomaterials.

Domain	Study	Chitosan System/Comparator	Negative, Neutral, or Cautionary Finding	Formulation-Dependent Lesson
Osteochondral scaffold repair	Roffi et al. [58]	Magnesium-doped hydroxyapatite/collagen/chitosan composite scaffold	The composite chitosan-reinforced scaffold failed to provide superior osteochondral regeneration in preclinical models.	Adding chitosan to a multiphasic scaffold does not guarantee osteochondral repair; degradation, integration, residual scaffold, cartilage layer formation, and subchondral bone remodeling must be optimized together.
Large-animal cartilage repair	Bell et al. [59]	Rapidly degrading pre-solidified 10 kDa chitosan–blood implant vs. blood implant control in sheep critical-size chondral defects	The implant did not improve cartilage resurfacing compared with blood implant control. Repair tissue remained inferior to intact cartilage, with approximately 2-fold lower GAG and fibril modulus and approximately 4.5-fold higher permeability.	Molecular weight, degradation rate, species, defect size, and marrow-stimulation geometry strongly influence chitosan–blood implant performance; rabbit findings should not be directly extrapolated to large-animal defects.
Minipig cartilage repair	Hede et al. [128]	CARGEL Bioscaffold plus bone marrow stimulation vs. bone marrow stimulation alone	CARGEL increased fibrocartilage proportion, 80% vs. 64%, but hyaline cartilage was seen in only one treated defect and no broad superiority was observed across ICRS II parameters.	Increased defect filling or fibrocartilage formation is not equivalent to hyaline cartilage regeneration; repair-tissue composition must be interpreted carefully.
Clinical knee osteochondral lesions	Sofu et al. [130]	Chitosan–glycerol phosphate/blood implant vs. hyaluronic acid-based cell-free scaffold	Clinical and radiographic outcomes were similar between the chitosan-based implant and hyaluronan scaffold groups.	Chitosan augmentation may be clinically useful but is not automatically superior to other scaffold-based marrow-stimulation adjuncts.
Clinical talus cartilage repair	Camurcu et al. [63]; Akmeşe et al. [64]	Microfracture plus chitosan–blood implant vs. microfracture alone; chitosan scaffold vs. hyaluronan scaffold	Comparative talus studies did not show consistent superiority of chitosan for global clinical or MOCART outcomes; VAS function favored chitosan in one study, but broader superiority was not established.	Joint-specific translation matters. Knee BST-CarGel evidence should not be generalized automatically to talar osteochondral lesions.
Critical-size bone defects	Oryan et al. [60]	Chitosan, gelatin, chitosan–gelatin, autograft, and empty control in rat radial critical-size defects	Chitosan alone did not promote considerable new bone formation, whereas gelatin and chitosan–gelatin improved new bone formation and mechanical properties.	Chitosan alone may be insufficient for bone regeneration; composite phases, gelatin/collagen blending, mineral reinforcement, or bioactive functionalization are often required.
Small-molecule bone scaffold delivery	Agnes et al. [264]	BIO-encapsulated chitosan-based scaffold in mouse femoral critical-size defect	BIO scaffold reduced the distance between bone ends and increased vascularity, but complete bony bridging and significantly higher bone volume were not achieved.	Partial regenerative signals should not be overinterpreted as complete bone repair; dose, release kinetics, scaffold mechanics, and angiogenesis–osteogenesis coupling require optimization.
Anti-infective porous titanium coating	Croes et al. [205]	Chitosan–silver and chitosan–vancomycin coatings on additively manufactured porous titanium	Strong in vitro antibacterial activity, including up to 4-log reduction, did not translate into equivalent in vivo benefit for chitosan–silver; silver coatings aggravated infection-mediated bone remodeling.	In vitro bacterial killing is insufficient. Infection-control systems must be tested with host bone response, osteolysis, osteoclast activity, inflammation, and osseointegration.
Acrylic bone cement	Tunney et al. [52]	Chitosan incorporated into gentamicin-loaded Palacos R PMMA cement	Chitosan decreased gentamicin release, did not improve prevention of bacterial colonization or biofilm formation, and reduced compressive and bending strengths below ISO requirements after saline aging.	Chitosan can impair cement performance when poorly matched to PMMA formulation; antibiotic elution, aging, fatigue, and ISO-relevant mechanical safety must be tested before translation.
Acrylic bone cement bioactivity	Valencia Zapata et al. [228]	Acrylic bone cement with low-to-moderate chitosan loading	Chitosan improved bioactivity and reduced exotherm, but higher loading increased residual monomer and decreased mechanical properties.	Improved bioactivity can come at the expense of mechanical safety; chitosan-loaded acrylic cement may be more appropriate for lower-load indications unless mechanics are preserved.
Intervertebral disk hydrogel comparison	Naqvi et al. [263]	Alginate vs. chitosan hydrogels for nucleus pulposus cells and bone marrow stem cells	Nucleus pulposus cells survived in both hydrogels, but bone marrow stem cell viability was reduced in chitosan, and alginate supported greater sulfated GAG accumulation and collagen II deposition.	Chitosan is not universally superior to other hydrogels; disk applications require cell-type-specific and matrix-specific hydrogel selection.
Rotator cuff patch augmentation	Willbold et al. [243]	Electrospun chitosan-coated PCL graft vs. comparator repair strategies in rat infraspinatus model	The chitosan-coated graft showed cellular and vascular ingrowth but did not provide biomechanical superiority at 8 weeks.	Biological integration does not necessarily translate into mechanical repair strength; tendon-to-bone systems require load-to-failure, stiffness, cyclic loading, and failure-mode assessment.

**Note:** These studies were selected because they define formulation boundaries, comparator limitations, or translational risks. Their inclusion is intended to prevent overgeneralization of positive findings and to emphasize that chitosan performance depends on chemistry, architecture, degradation, payload, release kinetics, mechanics, host environment, and indication-specific testing.

## Data Availability

No new experimental data were generated in this study. The extracted literature data supporting this review are provided in the Supplementary Materials, including the master evidence table of the final evidence base of 258 unique publications and the application-specific evidence, reporting, translational-priority, and endpoint checklists.

## Supplementary Materials

The following supporting information can be downloaded at: https://www.mdpi.com/article/10.3390/polym18131644/s1, Table S1: Master evidence table of the final evidence base of 258 unique publications; Table S2: Detailed application-specific evidence tables; Table S3: Minimum reporting checklist for orthopedic chitosan biomaterials; Table S4: Priority clinical and translational studies; Table S5: Application-specific endpoint checklist; Table S6: Search strategy, databases, conceptual blocks, and representative PubMed/MEDLINE search strings.

## Abbreviations

ACL	anterior cruciate ligament
ADLs	activities of daily living
AgNPs	silver nanoparticles
ALP	alkaline phosphatase
AOFAS	American Orthopedic Foot and Ankle Society score
BMD	bone mineral density
BMP-2	bone morphogenetic protein 2
BMSC	bone marrow-derived mesenchymal stem cell
BSP	bone sialoprotein
BST-CarGel	chitosan–glycerophosphate/whole-blood cartilage repair implant (trade name)
BV/TV	bone volume/total volume
CAHA	carboxymethyl chitosan/alginate/hydroxyapatite scaffold
CFU	colony-forming unit
CMCS	carboxymethyl chitosan
COL1	type I collagen
COL2 (*COL2A1*)	type II collagen (collagen type II alpha 1 chain)
COL3	type III collagen
CPC	calcium phosphate cement
CS	chitosan
CT	computed tomography
DAFM	decellularized annulus fibrosus matrix
DCT	dextran–chitosan–teleostean hydrogel
DHI	disk height index
DNA	deoxyribonucleic acid
ECM	extracellular matrix
FAK	focal adhesion kinase
GAG	glycosaminoglycan
GDF5	growth/differentiation factor 5
GelMA	gelatin methacryloyl
GO	graphene oxide
GP	glycerophosphate
HA	hyaluronic acid
HACC	hydroxypropyltrimethyl ammonium chloride chitosan
HAp	hydroxyapatite
HMT	hydroxyapatite microtube
HOS	Hip Outcome Score (ADL and sport-specific subscales)
ICRS	International Cartilage Repair Society
iHOT-33	International Hip Outcome Tool—33
IKDC	International Knee Documentation Committee score
IL	interleukin
ISO	International Organization for Standardization
KGN	kartogenin
KOOS	Knee Injury and Osteoarthritis Outcome Score
MC3T3-E1	murine preosteoblast cell line
MeGC	methacrylated glycol chitosan
MMP	matrix metalloproteinase
MOCART	Magnetic Resonance Observation of Cartilage Repair Tissue score
MRI	magnetic resonance imaging
MRSA	methicillin-resistant *Staphylococcus aureus*
MRSE	methicillin-resistant *Staphylococcus epidermidis*
MSC	mesenchymal stem cell
MSSA	methicillin-susceptible *Staphylococcus aureus*
NAHS	Non-Arthritic Hip Score
nano-HAp	nano-hydroxyapatite
NF-κB	nuclear factor kappa B
NP	nucleus pulposus
NPSC	nucleus pulposus stem cell
OA	osteoarthritis
OCN	osteocalcin
OPN	osteopontin
OST	oxidized starch
PBS	phosphate-buffered saline
PCL	polycaprolactone
PDA	polydopamine
PDI	polydispersity index
PEG	poly(ethylene glycol)
PEGDA	poly(ethylene glycol) diacrylate
PET	polyethylene terephthalate
PHBV	poly(3-hydroxybutyrate-co-3-hydroxyvalerate)
PLA	polylactic acid
PLGA	poly(lactic-co-glycolic acid)
PLLA	poly(L-lactic acid)
PMMA	poly(methyl methacrylate)
PRISMA	Preferred Reporting Items for Systematic Reviews and Meta-Analyses
PRISMA-ScR	PRISMA extension for Scoping Reviews
PROMs	patient-reported outcome measures
PRP	platelet-rich plasma
PTMC	poly(trimethylene carbonate)
PVA	poly(vinyl alcohol)
QCS	quaternized chitosan
RCT	randomized controlled trial
RGD	arginine–glycine–aspartate (Arg-Gly-Asp) peptide motif
rhBMP-2	recombinant human bone morphogenetic protein 2
ROS	reactive oxygen species
RUNX2	runt-related transcription factor 2
SANRA	Scale for the Assessment of Narrative Review Articles
SCX	scleraxis
SDF-1	stromal cell-derived factor 1
SEM	scanning electron microscopy
SF	silk fibroin
SOX9	SRY-box transcription factor 9
TCP	tricalcium phosphate
TDSC	tendon-derived stem cell
TGF-β	transforming growth factor beta
TNMD	tenomodulin
TSPC	tendon stem/progenitor cell
VAS	visual analog scale
VCM	vancomycin
VEGF	vascular endothelial growth factor
VRSA	vancomycin-resistant *Staphylococcus aureus*
WOMAC	Western Ontario and McMaster Universities Osteoarthritis Index
XRD	X-ray diffraction
ZnO	zinc oxide
